# A transient RNP compartment boosts translation and growth of mammalian oocytes

**DOI:** 10.1126/sciadv.aej5359

**Published:** 2026-08-28

**Authors:** Noemi Zollo, Gabriele Zaffagnini, Ali Haidar, Alexis Canette, Gaëlle Letort, Christelle Da Silva, Elsa Labrune, Nicolas Tessandier, Julien Dumont, Corinne Blugeon, Benoit Wattellier, Elvan Böke, Christopher Thomas, Maria Almonacid, Marie-Hélène Verlhac

**Affiliations:** ^1^ Center for Interdisciplinary Research in Biology (CIRB), Collège de France, Université PSL, CNRS, INSERM, 75005 Paris, France.; ^2^ Centre for Genomic Regulation (CRG), The Barcelona Institute of Science and Technology, Barcelona, Spain.; ^3^ Sorbonne Université, CNRS, Inserm, Institut de Biologie Paris-Seine (IBPS), 75005 Paris, France.; ^4^ Department of Developmental and Stem Cell Biology, Institut Pasteur, CNRS UMR 3738, Université Paris Cité, 25 rue du Dr. Roux, 75015 Paris, France.; ^5^ Hospices Civils de Lyon, Inserm U1208, SBRI, 59 bd Pinel, 69500 Bron, France.; ^6^ Université Claude Bernard Lyon 1, Faculté de médecine Laennec, 7 rue Guillaume Paradin, 69008 Lyon, France.; ^7^ GenomiqueENS, Institut de Biologie de l’ENS (IBENS), Département de Biologie, École Normale Supérieure, CNRS, INSERM, Université PSL, 75005 Paris, France.; ^8^ Phasics, Espace Technologique, 91190 Saint Aubin, France.; ^9^ Institució Catalana de Recerca i Estudis Avançats (ICREA), Barcelona, Spain.; ^10^ Aix Marseille University, CNRS, IBDM, Turing Centre for Living Systems, 13288 Marseille, France.

## Abstract

Mammalian oocytes undergo an extended growth phase, during which transcription gradually declines. To sustain meiotic divisions and early embryonic development, growing oocytes must accumulate maternal stores and tightly regulate translation. Using immunofluorescence, mass spectrometry, electron microscopy, RNA sequencing, and live ex vivo ovarian tissue–slice imaging, we identified a transient ribonucleoprotein-organelle compartment in growing mouse oocytes, which we named the Zollo body. Although it resembles the Balbiani body observed in nongrowing oocytes of many vertebrate species, the Zollo body arises during growth and shows nascent translation and phospho-mTOR enrichment. Pharmacological dissolution of the Zollo body arrests follicle and oocyte growth in cultured ovarian tissue slices. Thus, the Zollo body is a transient, translation-associated compartment required for normal follicle and oocyte growth.

## INTRODUCTION

Female fertility is increasingly at risk in modern societies, often due to poor oocyte quality. Oocytes are exceptionally large cells, and their developmental potential depends heavily on the successful accumulation of maternal proteins, RNAs, lipids, and metabolites during their growth in the ovary. In mice, oocytes rely almost entirely on translational regulation during the final stages of growth, when their size increases rapidly despite a marked reduction in transcriptional activity (*1*–*3*). The developmental potential of the oocyte thus depends on its ability to accumulate maternal mRNAs into ribonucleoprotein (RNP) complexes and precisely regulate their translation. In most vertebrates, including humans, a well-characterized structure known as the Balbiani body is transiently assembled in nongrowing oocytes during the earliest stages of oogenesis, before the growth phase. The Balbiani body contains RNAs and RNPs, as well as endoplasmic reticulum (ER), Golgi apparatus, and mitochondria, and is thought to preserve these organelles and transcripts during early oogenesis (*4*, *5*). Proteomic profiling of the Balbiani body in *Xenopus* and zebrafish has revealed a set of shared proteins, including highly conserved factors involved in the sequestration of translationally silent mRNAs (*6*, *7*). The assembly of the Balbiani body in these two species is driven by a prion-like protein (Xvelo/Bucky Ball homologs, respectively) capable of forming an amyloid-like matrix that can reversibly trap organelles and RNPs (*6*, *8*). In both *Xenopus* and zebrafish, Xvelo/Bucky Ball proteins direct the incorporation of germline mRNAs into the Balbiani body, promoting their insolubilization and thereby reducing their susceptibility to degradation (*9*). Fully grown mouse oocytes have also been shown to form a membraneless condensate [mitochondrial-associated ribonuclear domain (MARDO)], which is assembled through the RNP protein ZAR1 and functions to silence maternal mRNAs (*10*). While a structure assembled inside a Golgi ring but devoid of mitochondria is present in primordial mouse oocytes, so far, no other higher-order condensate identical to the Balbiani body from other species has been observed in these oocytes (*11*–*13*). We describe a previously unidentified compartment, the Zollo body (Zb), that is transiently assembled in growing mouse oocytes present in secondary to early antral follicles. Its morphology is reminiscent of the Balbiani body, being highly enriched in RNAs, RNPs, mitochondria, Golgi, and ER; however, the Zb is distinct, arising much later in oogenesis, although still before the formation of the MARDO (*10*). Using immunofluorescence, electron microscopy, RNA sequencing (RNA-seq), and mass spectrometry, we systematically characterized the components and overall organization of this compartment. Dry-mass measurements of growing oocytes with or without this structure provided valuable insights into its functional role. Despite its morphological similarity to the Balbiani body, we find that the Zb is translation-associated, showing nascent translation and enrichment of phospho–mechanistic target of rapamycin (mTOR). Moreover, DDX5 inhibitor supinoxin rapidly dissolves the Zb and arrests follicle and oocyte growth in cultured ovarian tissue slices. Our results support a key role for this compartment in enhancing translation and cell growth while maintaining cytoplasmic density during oogenesis, thereby preventing cytoplasmic dilution, a process detrimental to cell fitness (*14*, *15*).

## RESULTS

### A previously unidentified compartment reminiscent of the Balbiani body is present in growing oocytes

**Fig. 1.**
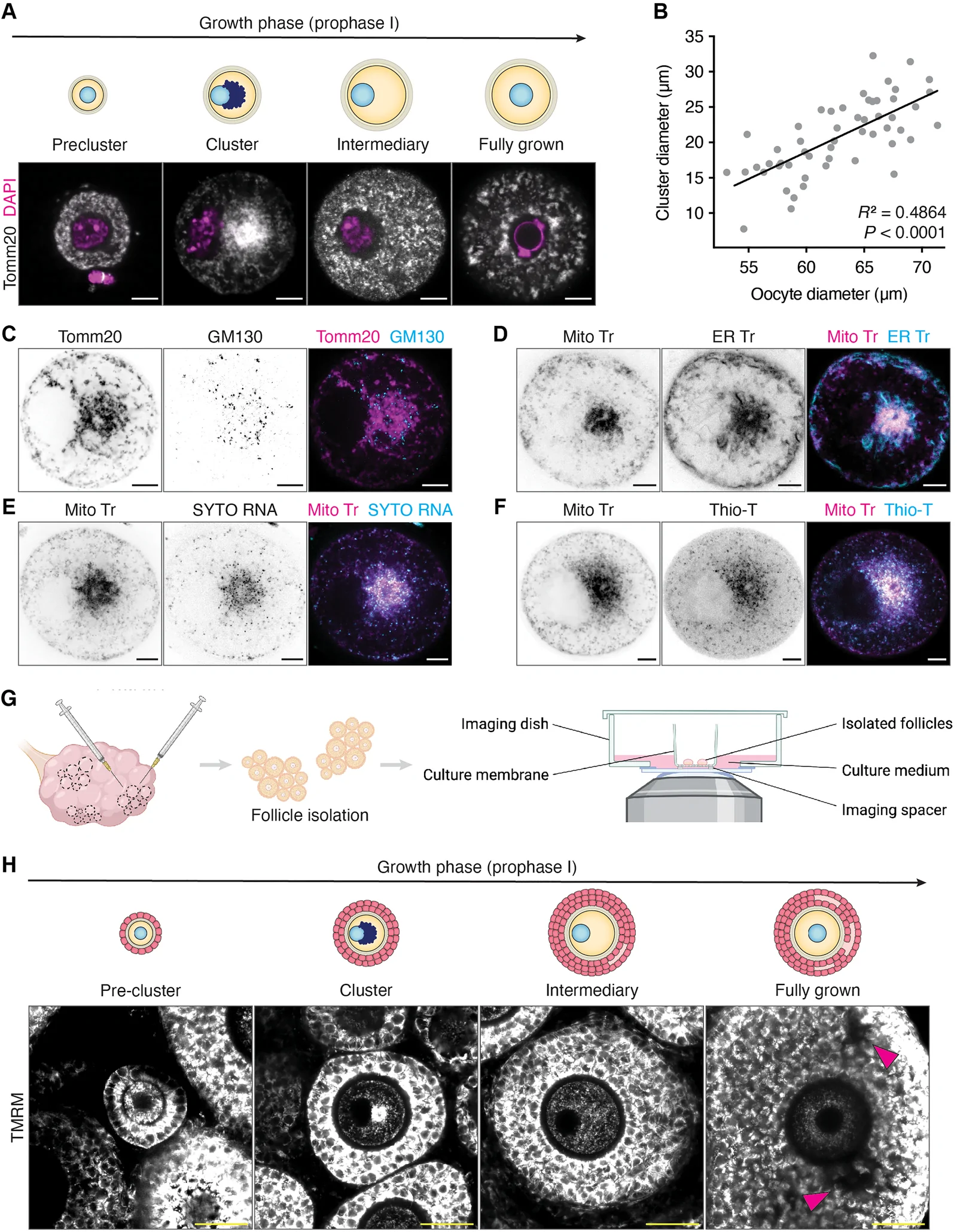
Growing mouse oocytes contain a previously unidentified cluster of organelles and RNPs. (**A**) Immunofluorescence staining of mouse oocytes displaying mitochondria labeling (TOMM20, white) at different stages of the end of the follicular growth phase, sorted according to their size and chromatin state [4′,6-diamidino-2-phenylindole (DAPI), pink] as in (*3*). (**B**) Quantification of cluster over oocyte diameter, measured at their maximal cross section in cluster stage oocytes. *n* = 53 oocytes for both cluster and oocyte measurements; three independent experiments. (**C**) Immunofluorescent staining indicating enrichment of Golgi (GM130, middle) in the cluster, identified by labeling mitochondria (TOMM20, left). The right panel shows the merge of mitochondria (pink) and Golgi (blue) staining. (**D**) Cluster stage oocyte stained with MitoTracker (Mito Tr; left) and ER Tracker (ER Tr; middle). The right panel shows the merge of Mito Tr (pink) and ER Tr (blue) staining. (**E**) Cluster stage oocyte stained with Mito Tr (left) and SYTO RNA Select (SYTO RNA; middle) to detect RNA localization. The right panel shows the merge of Mito Tr (pink) and SYTO RNA (blue) staining. (**F**) Cluster stage oocyte stained with Mito Tr (left) and thioflavin-T (Thio-T; middle) to reveal the presence of beta-amyloid fibrils. The right panel shows the merge of Mito Tr (pink) and Thio-T (blue) staining. (**G**) Schematic representation of the mechanical isolation of groups of secondary follicles from young mouse ovaries for live imaging. (**H**) Representative images of mouse growing oocytes within their follicle at different stages, stained with TMRM. The Zb is visible in the oocyte at the cluster stage, within a secondary follicle. Pink arrowheads highlight the formation of the follicular antrum. Scale bars, 10 μm (A and C to F) and 50 μm (H).

Oocytes retrieved from 3- to 4-week-old mice were classified on the basis of their size and chromatin state following the methodology described in (*3*) and were also sorted based on their zona pellucida thickness (Fig. 1A and fig. S1, A and B). We identified a local accumulation of mitochondria in oocytes with an average diameter of 65 μm, corresponding to a developmental stage we termed the cluster stage, which precedes the intermediary and fully grown stages (Fig. 1A) (*3*). This cluster of mitochondria forms adjacent to the nucleus and displays a range of sizes proportional to the oocyte diameter (Fig. 1B). This structure was not observed at later stages of oocyte growth, suggesting that it is a transient feature that disappears as the oocyte grows (Fig. 1A). This transient cluster is present in a subpopulation of growing oocytes, with a prevalence varying according to the age of the animal (fig. S1C). An accumulation of mitochondria adjacent to the nucleus has been recently observed by another group upon overexpression of TRAK2, a microtubule motor/outer mitochondrial membrane adaptor, although it has not been further characterized (*16*). We could also detect a local enrichment of mitochondria adjacent to the nucleus in some human oocytes at a stage of growth comparable to the cluster stage in the mouse (fig. S1D, left), which disappeared at later stages (fig. S1D, right) (*17*), suggesting that a similar organization may be conserved across mammals. Other membranous organelles such as Golgi (Fig. 1C) and ER (Fig. 1D) are enriched in this compartment, reminiscent of the Balbiani body from other species (*18*). Furthermore, the cluster is enriched in RNAs (Fig. 1E) and shows a positive staining for thioflavin-T (Fig. 1F), indicating the presence of amyloid fibers, a hallmark of proteins containing prion-like domains (PrLD) (*6*)*.* Similar to the Balbiani body in zebrafish (*19*), microtubules appear to contribute to the structural integrity of the cluster as treatment with nocodazole resulted in its partial disassembly (fig. S2A, left, and E to G). By contrast, F-actin is not involved in its maintenance because treatment with cytochalasin D did not induce its disassembly (fig. S2A, middle). Microfilaments are not required for its formation either, as shown by the presence of this cluster in oocytes lacking the actin nucleator formin-2 (*20*, *21*), which are devoid of cytoplasmic F-actin (fig. S2A, right). An acentriolar microtubule organizing center (MTOC), positive for pericentrin and PLK4, resides at the center of the cluster (fig. S2, B and C) where microtubules are nucleated (fig. S2, C to E). Following nocodazole washout, the cluster progressively reassembled around the acentriolar MTOC, which is normally present at this stage (*22*), over the course of few hours (fig. S2, H and I). This observation supports a role for microtubules in the assembly of this compartment.

Together, our observations reveal the presence of a previously unidentified structure in growing mammalian oocytes, which we have named the Zb. Its composition resembles that of the Balbiani body, and its assembly and maintenance are partially dependent on microtubules.

### The Zb is present in oocytes of secondary to early antral follicles

Because we had detected the Zb in mechanically isolated oocytes, we wanted to confirm that it was also present in situ, within oocytes in their native follicular environment. To do so, we adapted the protocol developed to image ovulation in isolated antral follicles (*23*) to establish a live ovarian tissue–slice imaging assay (Fig. 1G and Materials and Methods). These tissue slices were isolated from prepubertal mice aged 16 to 21 days and contained many developing follicles, allowing us to quantitatively image both oocyte and follicle growth live using a fluorescent mitochondrial probe (Fig. 1H and Materials and Methods). Consistent with our observations in isolated oocytes, the Zb was detected transiently in cluster stage oocytes found in secondary to early antral follicles, but not before (primary follicles) or after (late antral follicles; see pink arrowheads pointing at the developing antrum in Fig. 1H). As with isolated oocytes, the size of the Zb was variable (fig. S3A) and correlated with oocyte size inside the follicle (fig. S3B). The prevalence of the Zb was maximal in secondary follicles (80 to 170 μm in diameter) and decreased with follicle development (fig. S3C). Live imaging of follicle growth allowed us to observe the dynamics of Zb growth and shrinkage over the course of a few days (movie S1 and fig. S3D). At higher spatial and temporal resolution, we could detect clumps of mitochondria being gathered inside the Zb (fig. S3E, see yellow arrowheads), increasing its size (fig. S3F). Globally, the cross-sectional area of Zb-containing oocytes increased linearly with follicle cross-sectional area, suggesting that the two processes might be coupled (fig. S3G).

This innovative live ovarian tissue–slice imaging assay establishes that the Zb is not an artefact of mechanical oocyte isolation but is present in oocytes within follicles in their native ovarian context. It also allowed us to determine more precisely the beginning and end of Zb appearance. Zb assembly initiates in oocytes from secondary follicles, reaches its full size in oocytes from early antral follicles, and is no longer observed in late antral stages, corresponding to intermediary and fully grown oocytes. These observations allowed us to confidently pursue further characterization of the Zb in isolated oocytes.

### Electron microscopy defines Zb ultrastructure and organelle enrichment

**Fig. 2.**
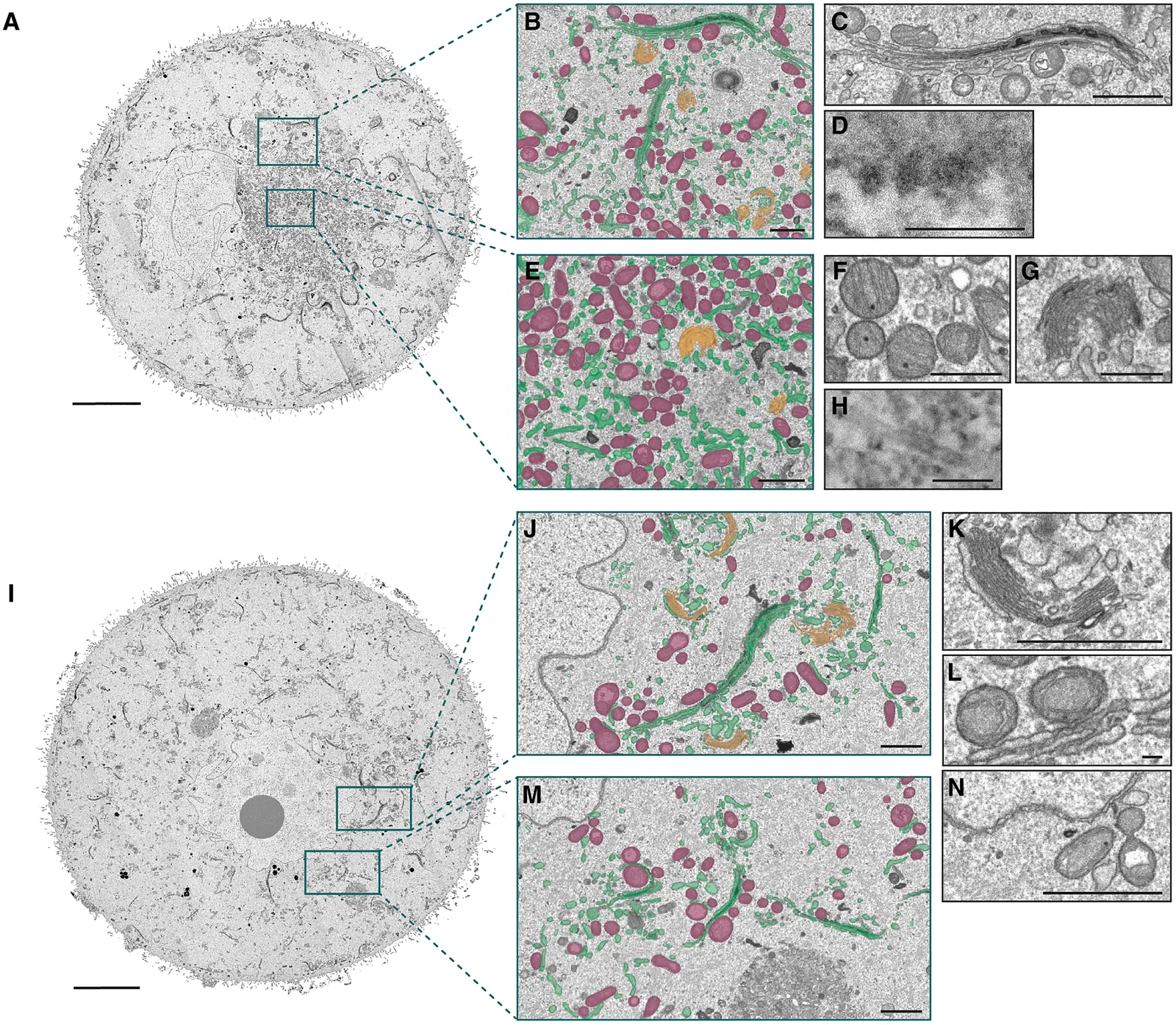
Electron microscopy confirms enrichment of organelles within the Zb. (**A**) Electron Microscopy image of a cluster stage oocyte and of (**I**) an intermediary stage oocyte; insets are magnifications of outlined regions. In (**B**), (**E**), (**J**), and (**M**), color coding was applied to mitochondria (purple), Golgi ministacks (orange), and ER (green). Insets (**C**) and (**D**) are magnifications from inset (B); insets (**F**), (**G**), and (**H**) are magnifications from inset (E); insets (**K**) and (**L**) are magnifications from inset (J); inset (**N**) is a magnification from inset (M). Scale bars, 10 μm (A and I), 1 μm (B, E, J, K, M, and N), 500 nm (C, F, and G), and 100 nm (D, H, and L).

To further characterize the internal organization of the Zb, we performed electron microscopy on isolated oocytes at the cluster and intermediary stages (see Materials and Methods). Consistent with live imaging and immunofluorescence, the Zb was observed adjacent to the nucleus, near the oocyte center (Fig. 2A). The structure displayed a clear enrichment of ER (green in Fig. 2, B and E, and enlarged in Fig. 2C) and mini-Golgi stacks (orange in Fig. 2, B and E, and enlarged in Fig. 2G) and was highly enriched in mitochondria (purple in Fig. 2, B and E, and enlarged in Fig. 2F). Ribosomes could be detected along the ER membrane (Fig. 2D). The center of the Zb showed a region less dense in organelles (Fig. 2, A and E), as seen by live imaging and immunofluorescence (Fig. 1, C to F), where microtubules were observed (Fig. 2H). The Zb was no longer visible in intermediary oocytes (Fig. 2I), consistent with Fig. 1 (A and H), and the cytoplasm instead showed a more dispersed distribution of mitochondria (Fig. 2, J, M, and L), ER (Fig. 2, J, M, and L), and mini-Golgi stacks (Fig. 2, J and K), even in regions close to the nuclear envelope (Fig. 2N). Together, these EM data validate the overall architecture of the Zb and confirm its enrichment in mitochondria, Golgi, and ER, as observed by immunofluorescence.

### RNA-seq of the isolated Zb reveals enrichment of organelle- and folliculogenesis-related mRNAs

**Fig. 3.**
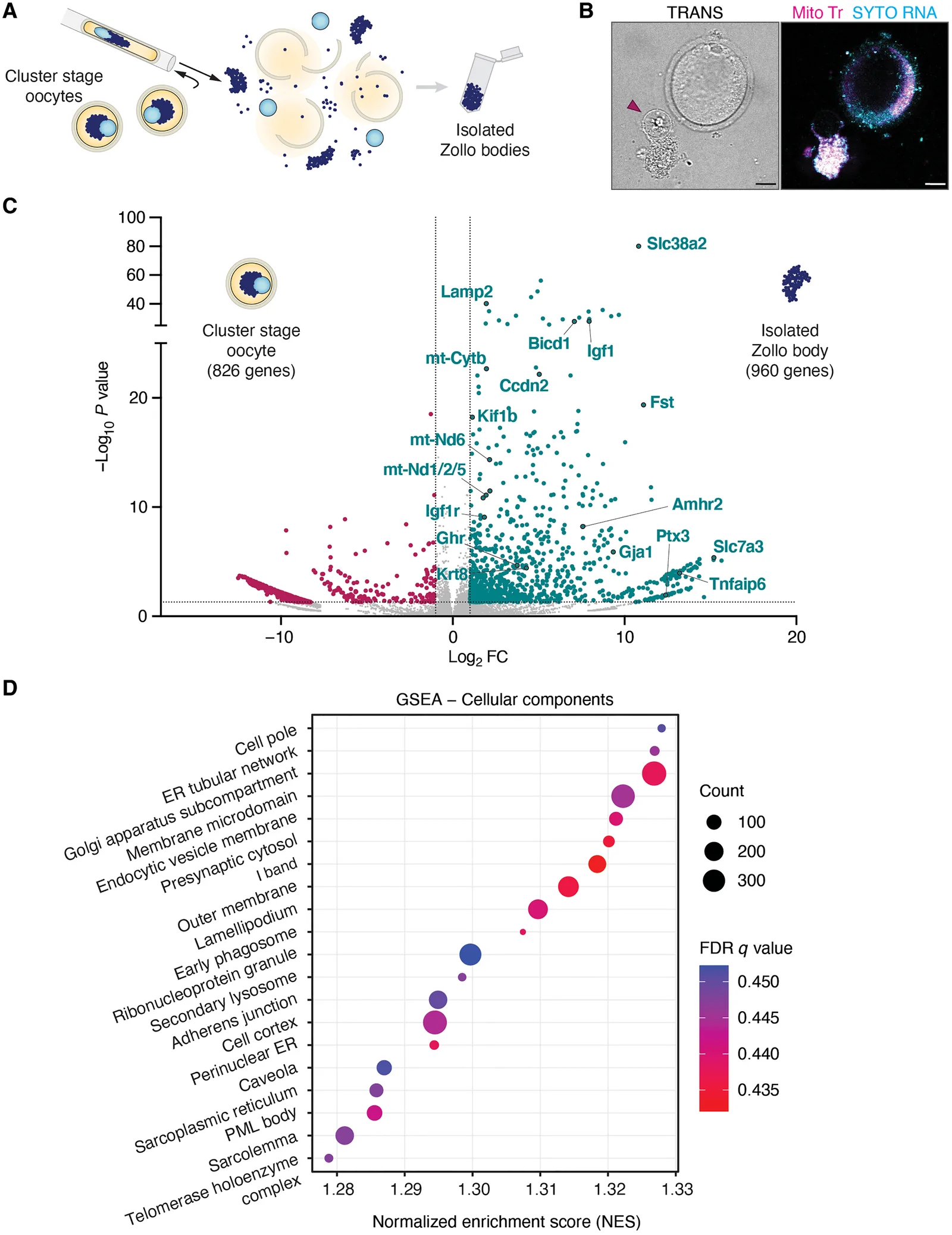
RNA-seq analysis of isolated Zbs allows the identification of compartment-specific genes. (**A**) Schematic representation of the Zb’s mechanical isolation for RNA-seq analysis. (**B**) Transmitted-light image of a mechanically isolated Zb and its corresponding remaining oocyte (TRANS; left), costained with Mito Tr (pink) and SYTO RNA (blue, right); the purple arrowhead indicates the nucleus attached to the cluster. (**C**) Volcano plot depicting differentially expressed genes in the isolated Zb versus whole cluster stage oocytes. A total of 826 down-regulated and 960 up-regulated genes (purple and green dots, respectively) were identified in the isolated Zb compared with whole oocytes, with an adjusted *P* value of <0.05 and average log_2_ fold change (FC) of <−1 or >1, respectively. (**D**) Gene set enrichment analysis (GSEA) of up-regulated genes in the isolated Zb showing enriched GO pathways in the category cellular components. PML, promyelocytic leukemia. Scale bars, 10 μm.

Because the Zb is enriched in RNAs (Fig. 1E), we sought to characterize their identity by mechanically isolating the Zb from cluster stage oocytes and performing poly(A)^+^ RNA-seq of the isolated Zb relative to whole oocytes (Fig. 3A). Upon isolation, the Zb remained stable for several hours outside the cell (Fig. 3B), similar to previous observations of the Balbiani body in *Xenopus* and zebrafish (*6*, *7*)*.* The sequencing identified 960 mRNAs significantly enriched inside the Zb (*P* < 0.05 with an average log_2_ fold change of >1) (Fig. 3C and table S1). Gene set enrichment analysis (GSEA) of Zb-enriched mRNAs revealed transcripts encoding components of organelles observed in the Zb (Fig. 3D), such as Golgi, mitochondria (*mt-Cytb*, *mt-Nd6*, and *mt-Nd1/2/5*, highlighted in Fig. 3C), ER, as well as lysosomes (whose presence in the Zb was confirmed in fig. S4A), and RNPs, suggesting that the Zb is enriched not only in these organelles but also in mRNAs encoding for proteins that compose them.

The Zb was also strongly enriched in mRNAs important for follicle development, including transcripts encoding follistatin (*Fst*), anti-Müllerian hormone receptor 2 (*Amhr2*), insulin-like growth factor 1 and its receptor (*Igf1* and *Igf1r*), growth hormone receptor (*Ghr*), cyclin D2 (*Ccnd2*), and connexin 43 (*Gja1*) (all highlighted in Fig. 3C). mRNAs encoding components of transforming growth factor–β signaling, a major pathway involved in folliculogenesis (*24*), are also enriched in the Zb (see the top “SMAD binding” category in the GSEA, fig. S4B). Because follicle development acquires increasing follicle-stimulating hormone (FSH; gonadotropin) dependence during the late preantral-to-early antral transition (*25*), it is notable that mRNAs encoding regulators and targets of gonadotropin signaling are accumulated in the Zb.

Consistent with the developmental timing of antrum formation, the Zb was also enriched in mRNAs encoding proteins associated with secretion (e.g., *Lamp2*) and amino acid transporters (*Slc38a2* or *Slc7a3*, highlighted in Fig. 3C). We also note enrichment of extracellular matrix–related mRNAs (“extracellular matrix binding,” “integrin binding,” and “fibronectin binding” categories in the GSEA; fig. S4B), potentially related to assembly of the extracellular matrix that maintains the cumulus-oocyte complex (*26*). mRNAs related to TSG-6 (*Tnfaip6*) and pentraxin 3 (*Ptx3*), proteins essential for organizing the extracellular matrix of the cumulus-oocyte complex, are also enriched in the Zb (highlighted in Fig. 3C) (*24*, *26*). Last, we detected enrichment of mRNAs encoding microtubule motors (*Kif1b* and *Bicd1*, highlighted in Fig. 3C), which could be related to the role of microtubules in Zb assembly and maintenance.

### Mass spectrometry reveals ribosome enrichment in the Zb

Based on the thioflavin-T staining results (Fig. 1F), we hypothesized that PrLD-containing proteins may contribute to the assembly of this compartment, analogously to the role of Xvelo/Bucky ball in the Balbiani body of *Xenopus* and zebrafish, respectively (*6*, *7*)*.*

**Fig. 4.**
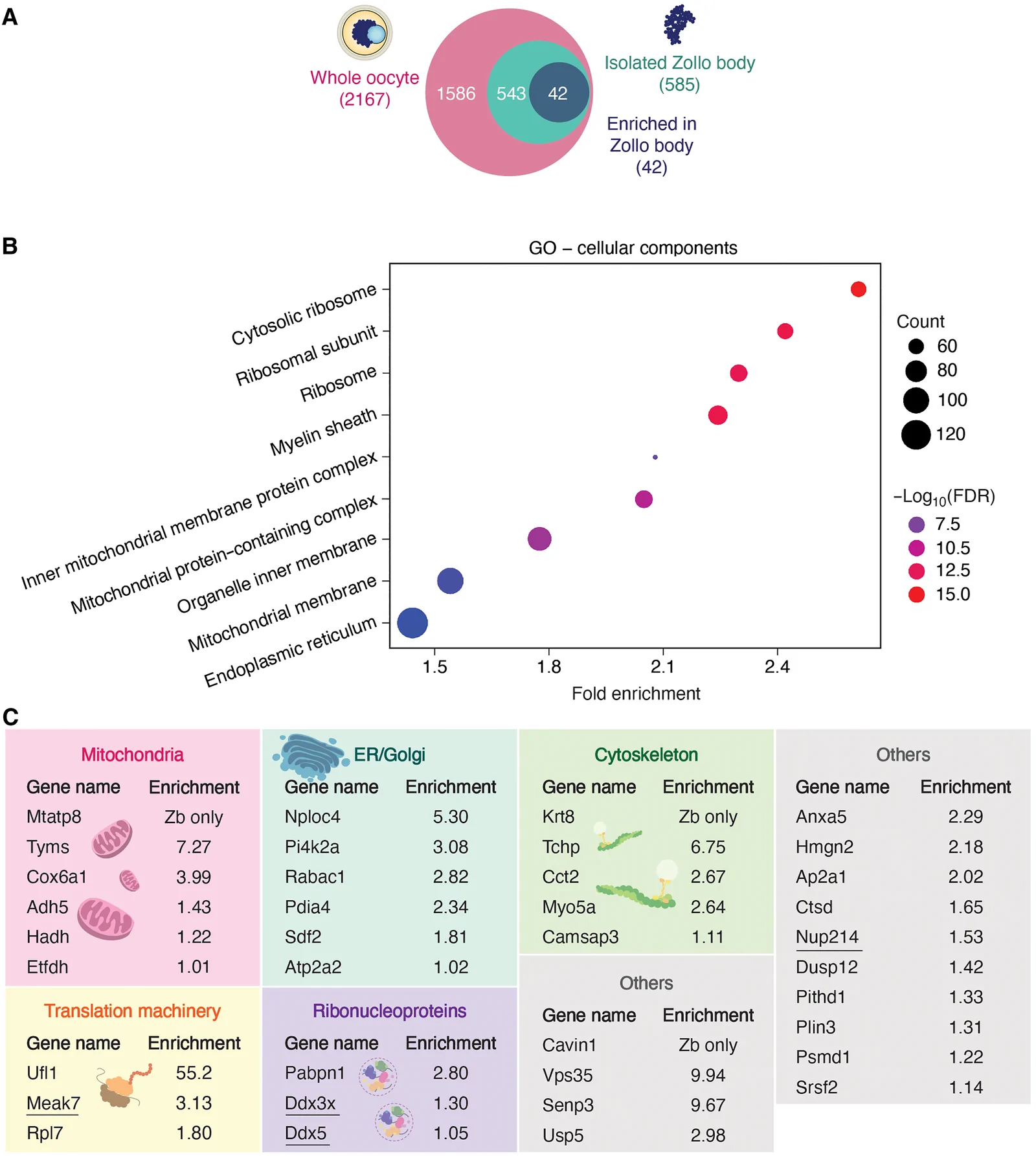
Mass spectrometry analysis of isolated Zbs reveals an enrichment in ribosomes. (**A**) Diagram showing relative proportions of proteins identified by mass spectrometry in the whole oocyte at the cluster stage (pink), in the isolated Zb (green) and enriched in the isolated Zb (blue). The numbers refer to the total number of proteins for each category (Whole oocyte, Isolated Zb and Enriched in the Zb). (**B**) Enriched Gene Ontology (GO) pathways for proteins identified in the isolated Zb. (**C**) List of the 42 proteins found enriched in the isolated Zb; each gene is shown with its corresponding enrichment value, obtained from the ratio of the abundance in the isolated Zb over the abundance in the whole oocyte at the cluster stage. Proteins with a PrLD are underlined.

We therefore aimed to identify the components responsible for the cohesion of the Zb and performed mass spectrometry on isolated Zbs to gain insight into its molecular composition. The mass spectrometry of isolated Zb compared with that of whole cluster stage oocytes identified 2167 proteins in oocytes and 585 in the isolated Zb, of which 42 proteins were significantly enriched in the Zb (Fig. 4A and table S2). Gene Ontology analysis of the 585 proteins identified in the Zb revealed an enrichment of ribosomal subunits, alongside the expected enrichment of proteins associated with membranous organelles and mitochondria (Fig. 4B). Examination of the 42 proteins enriched in the Zb (Fig. 4C) revealed multiple components of the translation machinery and a subset of proteins containing PrLDs (underlined in Fig. 4C and fig. S5A). We confirmed by immunofluorescence that DDX3X and DDX5 (two PrLD-containing helicases) are found within the Zb (fig. S5, A to C and I). Consistent with the idea that a PrLD-containing protein promotes Zb cohesion and contributes to its mechanical stability, DDX3X–green fluorescent protein (GFP) exhibited incomplete and slow fluorescence recovery after photobleaching within the Zb, whereas it recovered rapidly and completely in the surrounding cytoplasm (fig. S5, D and E). This result suggests that DDX3X may be a component of a structural amyloid matrix that contributes to the cohesiveness of the Zb.

While the Zb contained RNPs such as DDX3X and DDX5, it was not enriched for the canonical P-body marker LSM14A (fig. S5, F and I) (*27*, *28*). Using immunofluorescence, we further confirmed the enrichment of ribosomal proteins (fig. S5, G and I) and keratin 8 (fig. S5, H and I) in the Zb. When we compared the mRNAs significantly enriched in the Zb (*P* < 0.05) with the proteins detected in the Zb, we identified 39 common gene products, including KRT8, DDX5, and multiple ribosomal subunits (table S2, fourth sheet). This overlap suggests that, at least, a subset of poly(A)^+^ mRNAs may be translated within the Zb. Together, our findings show that the Zb is enriched in ribosomal and mitochondrial proteins and exhibits material properties consistent with a hydrogel-like compartment, reminiscent of material property of the Balbiani body.

### Multiple lines of evidence support active translation in the Zb

**Fig. 5.**
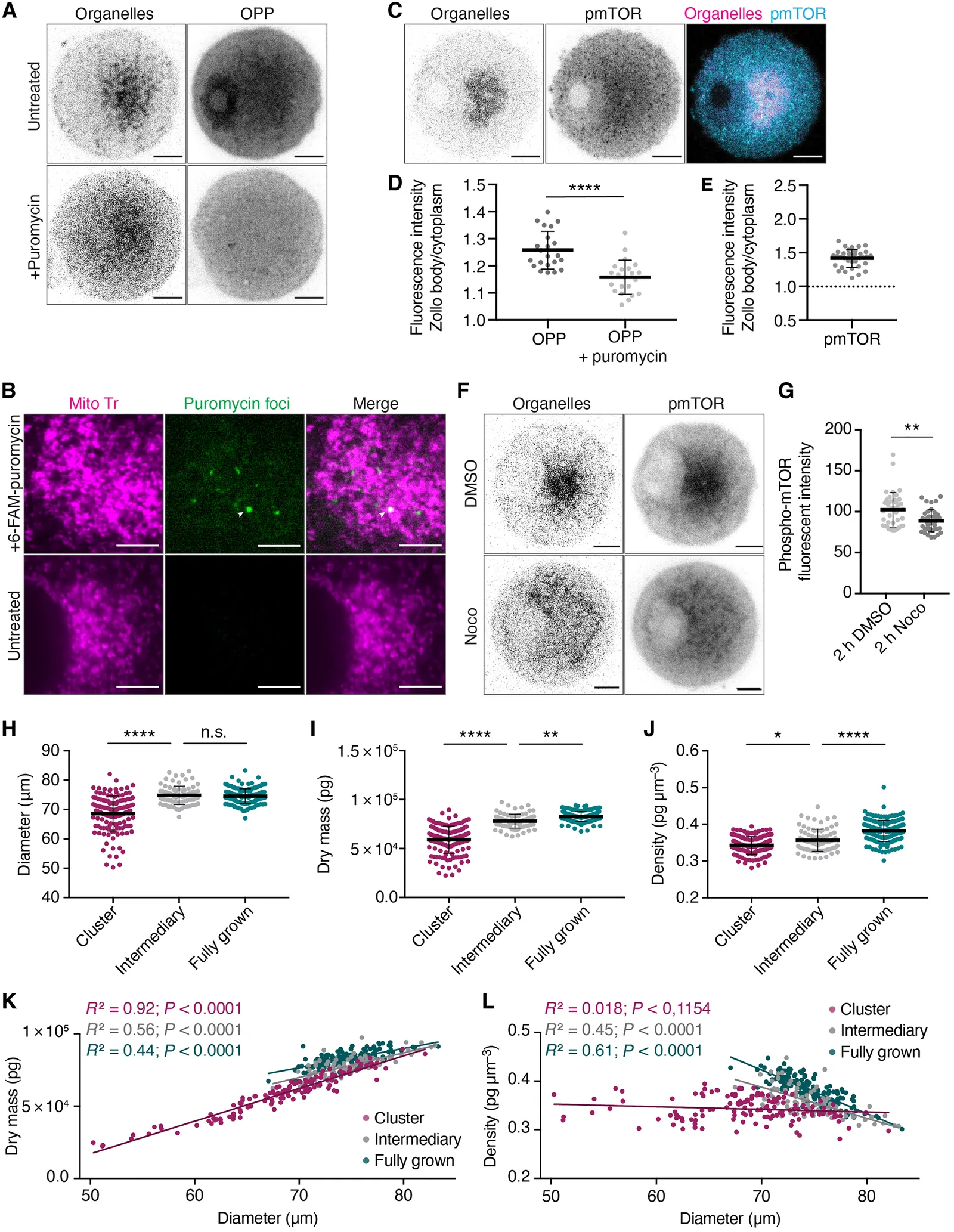
The Zb is associated with active translation. (**A**) Oocyte at the cluster stage stained for nascent translation by Click chemistry with the puromycin analog OPP (OPP, right column), negative control condition previously treated with puromycin for 1 hour (+puromycin, bottom row). The Zb is visualized through the autofluorescence of its organelles at 488 nm (organelles, left column). (**B**) Top row: live cluster stage oocyte labeled with 6-FAM-dC-puromycin (green) to reveal nascent peptides as fluorescent foci (arrowhead) in the Zb. Bottom row: control oocyte not exposed to 6-FAM-dC-puromycin. MitoTracker (magenta) highlights the Zb. (**C**) Cluster stage oocyte labeled with anti–phospho-mTOR Ser^2448^ (pmTOR; middle); the Zb is visualized through the autofluorescence of its organelles at 488 nm (organelles, left). The right panel shows the merge of organelles (pink) and pmTOR (blue) staining. (**D**) Quantification of nascent translation signal intensity at the cluster stage as in (A). *n* = 22 oocytes for OPP and *n* = 22 oocytes for OPP + puromycin; five independent experiments. (**E**) Quantification of Phospho-mTOR signal intensity at the cluster stage as in (C). *n* = 34 oocytes; four independent experiments. (**F**) Oocyte at the cluster stage labeled with anti–phospho-mTOR Ser^2448^, (right column) after 2 hours of treatment with nocodazole (Noco; bottom row) or its solvent DMSO (top row). The Zb is visualized by autofluorescence of its organelles at 488 nm (organelles, left column). (**G**) Quantification of fluorescence intensity at the cluster stage from (F). *n* = 39 oocytes for DMSO, 2 hours; *n* = 39 oocytes for nocodazole, 2 hours; five independent experiments. h, hours. (**H**) Oocyte diameter at the cluster, intermediary, and fully grown stages measured with quantitative phase imaging. *n* = 139 oocytes for cluster, *n* = 82 oocytes for intermediary, and *n* = 160 oocytes for fully grown; six independent experiments. (**I**) Oocyte dry mass at the cluster, intermediary, and fully grown stages measured with quantitative phase imaging. *n* = 139 oocytes for the cluster stage, *n* = 82 oocytes for the intermediary stage, and *n* = 160 oocytes for the fully grown stage; six independent experiments. (**J**) Oocyte protein density at the cluster, intermediary, and fully grown stages estimated from measurements in (H) and (I). *n* = 139 oocytes for cluster, *n* = 82 oocytes for intermediary, and *n* = 160 oocytes for fully grown; six independent experiments. (**K**) Simple linear regression of dry mass values from (I) plotted against diameter measurements from (H) for the cluster, intermediary, and fully grown stages. (**L**) Simple linear regression of density values from (J) plotted against diameter measurements from (H) for the cluster, intermediary, and fully grown stages. Data are shown as means ± SD. *P* values were calculated using unpaired two-tailed Student’s *t* test with Welch’s correction for (D), Mann-Whitney test for (G), or one-way ANOVA with Tukey’s post hoc test for (H), (I), and (J). Scale bars, 10 μm. n.s., not significant; *P < 0.05; **P < 0.01; ****P < 0.0001.

Because the Zb is enriched in ribosomes and poly(A)^+^ mRNAs encoding organelle-related proteins, we hypothesized that the cluster stage is associated with active translation. To test this, we first used a Click-iT assay using a fluorescent puromycin–based probe to detect nascent peptides (see Materials and Methods) (*29*)*.* We observed a significant accumulation of fluorescent labeling inside the Zb (Fig. 5A, top row), which was abrogated by addition of the nonfluorescent competitor puromycin, supporting signal specificity (Fig. 5A, bottom row, quantified in Fig. 5D). This observation provided direct evidence of active translation inside the Zb. To examine this in live oocytes, we used a fluorescent puromycin conjugate that incorporates into nascent peptides (see Materials and Methods) (*30*). With a shorter labeling time of 10 min (compared with 30 min for Click-iT), we detected fluorescent foci accumulating predominantly within the Zb, rather than in the surrounding cytoplasm (Fig. 5B, top row). These foci were absent in oocytes untreated with this conjugate (Fig. 5B, bottom row). This observation reproduces the result obtained with the Click-iT assay and is consistent with the Zb being a site of nascent peptide production.

Activation of the mTOR pathway, and, in particular, phosphorylation of mTORC1, has been shown to integrate various metabolic signals and promote ribosome biogenesis and protein synthesis, ultimately supporting cell growth (*31*). As a second line of evidence, we detected an enrichment of phospho-mTOR inside the Zb compared with the surrounding cytoplasm (Fig. 5C, quantified in Fig. 5E). Enrichment of active mTOR is consistent with SLC38A2-encoding mRNA being the most enriched transcript inside the Zb (∼1800-fold, Fig. 3C and table S1), because this amino acid transporter has been shown to activate mTOR and regulate cell osmolarity (*32*). Eventually, it is also consistent with the presence of lysosomes in the Zb (fig. S4A), shown to act as recruitment platforms for the mTOR pathway (*31*). The overall levels of activated mTORC1 decreased upon nocodazole treatment (Fig. 5F, quantified in Fig. 5G), consistent with a requirement for Zb integrity to sustain high translational activity at this stage.

As a third line of evidence, we measured the dry mass at the cluster, intermediary, and fully grown stages of oocyte growth. Results highlighted a substantial increase in oocyte diameter between the cluster and intermediary stages, whereas the increase was not significant between the intermediary and fully grown stages (Fig. 5H). Using quantitative phase imaging (Materials and Methods), we observed a corresponding substantial increase in dry mass between the cluster and intermediary stages (Fig. 5I). Dry mass is a useful proxy to estimate the levels of protein synthesis, because proteins constitute ∼60% of the dry mass of mammalian cells (*33*). Only cluster stage oocytes were sensitive to a reduction in global protein synthesis during a 5-hour puromycin treatment, showing a modest but significant decrease in size and dry mass (fig. S6, A to C). While puromycin, which blocks protein translation, displayed a modest effect, a 5-hour nocodazole treatment had no significant impact on either parameter at the cluster stage (fig. S6, D to F). Together, these results argue for sustained protein synthesis between the cluster and intermediary stages. In a context of sustained growth, maintaining cytoplasmic density becomes essential to prevent deleterious cytoplasmic dilution that would compromise cell fitness (*14*, *34*). Accordingly, protein density did not decrease between the cluster and intermediary stages despite the significant increase in cell size (Fig. 5J). While dry mass increases linearly with oocyte diameter across the three stages (Fig. 5K), quantitative phase imaging revealed that the cluster stage is distinct in terms of protein density. Compared with the later stages, cluster stage oocytes maintain a constant cytoplasmic density across oocyte sizes, indicating tighter control over protein content (Fig. 5L). Together, these observations support active translation inside the Zb, boosting oocyte growth as transcriptional activity gradually declines.

### DDX5 inhibition dissolves the Zb and arrests follicle and oocyte growth

**Fig. 6.**
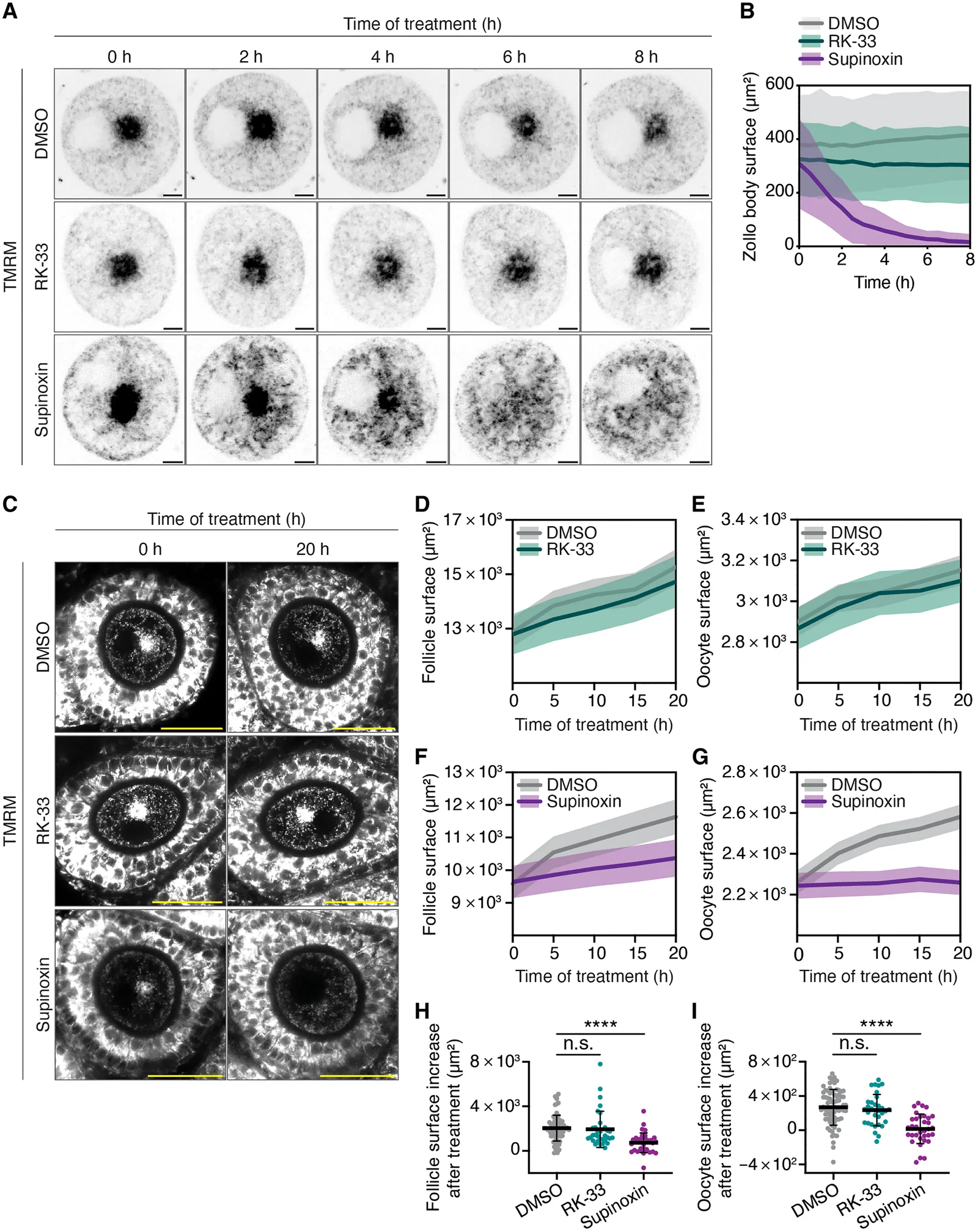
An inhibitor of DDX5 activity, supinoxin, dissolves the Zb and arrests oocyte and follicle growth. (**A**) Representative images of isolated cluster stage oocytes stained with TMRM and treated with DMSO (top row), 10 μM RK-33 (middle row), or 200 nM supinoxin (bottom row) followed in live. (**B**) Quantification of the Zb surface in isolated oocytes at the cluster stage treated with DMSO, 10 μM RK-33, or 200 nM supinoxin for 8 hours. *n* = 25 oocytes for DMSO, *n* = 26 oocytes for RK-33, and *n* = 39 oocytes for supinoxin; three independent experiments. (**C**) Representative images of cluster stage oocytes in their follicles, stained with TMRM and treated with DMSO (top row), 10 μM RK-33 (middle row), or 200 nM supinoxin (bottom row) at different time points. h, hours. (**D**) Quantification of follicle surface in isolated groups of secondary follicles (see fig. S3) treated with DMSO or 10 μM RK-33 for 20 hours. *n* = 43 follicles for DMSO and *n* = 33 follicles for RK-33; two independent experiments. (**E**) Quantification of oocyte surface in isolated groups of secondary follicles (see fig. S3) treated with DMSO or 10 μM RK-33 for 20 hours. *n* = 46 follicles for DMSO and *n* = 33 follicles for RK-33; two independent experiments. (**F**) Quantification of follicle surface in isolated groups of secondary follicles (see fig. S3) treated with DMSO or 200 nM supinoxin for 20 hours. *n* = 31 follicles for DMSO and *n* = 36 follicles for supinoxin; two independent experiments. (**G**) Quantification of oocyte surface in isolated groups of secondary follicles (see fig. S3) treated with DMSO or 200 nM supinoxin for 20 hours. *n* = 29 follicles for DMSO and *n* = 34 follicles for supinoxin; two independent experiments. (**H**) Quantification of follicle surface increase after 20 hours of treatment with DMSO, 10 μM RK-33, or 200 nM supinoxin. *n* = 71 follicles for DMSO, *n* = 36 follicles for RK-33, and *n* = 33 follicles for supinoxin; two independent experiments. (**I**) Quantification of oocyte surface increase within follicles after 20 hours of treatment with DMSO, 10 μM RK-33, or 200 nM supinoxin. *n* = 72 follicles for DMSO, *n* = 34 follicles for RK-33, and *n* = 33 follicles for supinoxin; two independent experiments. Data are shown as means ± SD. *P* values were calculated using Mann-Whitney test for (H) and unpaired two-tailed Student’s *t* test with Welch’s correction for (I). Scale bars, 10 μm (A) and 50 μm (C). n.s., not significant; ****P < 0.0001.

Because the Zb was positive for thioflavin-T and contained proteins with PrLDs, we hypothesized that PrLD-containing proteins may be important for its integrity, analogous to Xvelo/Bucky ball in the Balbiani body of *Xenopus* and zebrafish, respectively (*6*, *7*). To test this, we used inhibitors of DDX3X and DDX5, two helicases present in the Zb, on isolated oocytes stained with a fluorescent mitochondrial probe to visualize Zb dynamics. RK-33, a DDX3X inhibitor (*35*), induced a modest decrease in Zb cross-sectional area compared with dimethyl sulfoxide (DMSO)–treated controls (Fig. 6, A, middle, and B, green curve). In contrast, supinoxin, a DDX5 inhibitor (*36*), induced efficient dissolution of the Zb within ∼2 to 4 hours (Fig. 6, A, bottom, and B, purple curve). Supinoxin also induced Zb dissolution in oocytes within follicles, as tested using our live ovarian tissue–slice imaging assay, while RK-33 did not (Fig. 6C); mitochondria in the surrounding follicular cells were unaffected in both treatments. We imaged the treated ovarian tissue slices over a period of 20 hours and observed a pronounced increase in both follicle and oocyte cross-sectional area in DMSO- and RK-33–treated samples, which was suppressed in the supinoxin-treated group (Fig. 6, D to G, compare gray and green curves to purple curves). Ultimately, inhibition of DDX5 by supinoxin induced a highly significant reduction in follicle and oocyte growth compared with DMSO- or RK-33–treated samples (Fig. 6, H and I). Together, these data identify Zb integrity as a functional requirement for sustained oocyte and follicle growth, providing a direct link between this transient compartment and follicle development.

## DISCUSSION

The global architecture of the previously unidentified compartment characterized here in mouse oocytes, which we termed the Zb, is reminiscent of the Balbiani body from other vertebrate species. It contains RNAs, RNPs, mitochondria, Golgi, and ER and is organized around an MTOC that nucleates microtubules. The structure is maintained by a prion-like protein that may constitute an amyloid-like matrix, consistent with the reduced protein diffusion observed within the compartment. Despite these similarities, the Zb appears distinct from the Balbiani body. First, the Zb appears during oocyte growth while the Balbiani body is formed in earlier nongrowing oocytes (*18*)*.* Second, the Balbiani body has been associated with the storage of maternal mRNAs, as well as with the preservation of organelles in the context of oocyte dormancy (*4*, *5*, *37*). Similarly, the MARDO observed in fully grown mouse oocytes stores mRNAs and represses their translation (*10*). In contrast, we provide multiple lines of evidence indicating that the Zb is associated with active translation, in the context of sustained oocyte growth. It is remarkable that the Zb in mouse oocytes is a higher-order, mitochondria-, RNA-, and RNP-containing, membraneless compartment like the MARDO and the Balbiani body (*4*, *5*, *10*) yet emerges at a different stage of oogenesis. This is particularly interesting given that we noticed from published datasets that both the zebrafish and *Xenopus* Balbiani body also contain ribosomes and other translation-related factors (*6*, *7*). From an evolutionary point of view, the comparison between the Zb and the Balbiani body of other species is particularly fascinating as it suggests that a similar structure, composed of overlapping components, could perform different functions in a context-dependent manner.

Although it may seem counterintuitive for a hydrogel-like structure such as the Zb to be associated with an active process like translation, increasing evidence in the germline of several organisms supports a role for localized translation within RNP condensates. Examples of hydrogel-like condensates as translation activation sites have already been reported in *Drosophila*, where germ granules are sites for active translation (*38*), with their biphasic organization allowing a switch between mRNA repression at the core and translation at the surface (*39*). The Zb would therefore constitute an example of a higher-order condensate promoting translation.

The Zb might also be porous, containing microcompartments that allow free molecular diffusion. Confinement within such compartments could even enhance enzymatic reactions, with pores increasing the surface area available for these processes. In this context, mitochondria trapped within the matrix may not only be protected but could also serve as a source of adenosine 5′-triphosphate (ATP) for enzymatic reactions, especially considering that protein synthesis is the most ATP-consuming process in growing cells (*40*).

The translation-promoting function of the Zb could be of key importance during the growth phase of oogenesis, when transcription is progressively down-regulated (*1*–*3*). In this context, we propose that this structure could limit cytoplasmic dilution, helping to prevent senescence-like phenotypes reported in other cell types (*14*, *15*). It is intriguing that Zb size scales with oocyte size under physiological conditions, suggesting that this structure, which contains most of the cellular organelles, may adapt to the metabolic demands of the oocyte. Supporting this idea, the strong enrichment within the Zb of poly(A)^+^ mRNAs encoding proteins associated with Zb-resident organelles may indicate a role in organelle biogenesis. This hypothesis is consistent with the well-documented increase in membranous organelles during oocyte growth required to sustain cytoplasmic functionality (*41*). In particular, mitochondrial DNA copy number increases from ∼200 in mouse primordial follicles to more than 400,000 in fully grown oocytes, reflecting extensive mitochondrial proliferation (*42*). Further studies will be required to directly test the role of the Zb in organelle biogenesis.

Rodent homologs of Buc from zebrafish Balbiani body (*43*) and Xvelo from *Xenopus* (*6*), which form the amyloid matrix holding the Balbiani body, have not yet been identified (*43*). However, we identify several prion-like proteins within the Zb, including two ATP-dependent helicases DDX3X and DDX5, both involved in protein synthesis (*44*) and reported to form condensates in various physiological contexts (*45*, *46*). Both proteins have also been implicated in oogenesis and reported to function cooperatively (*47*–*49*). It has been shown in zebrafish that phase separation of the DDX3X homolog into condensates promotes the translation of essential maternal mRNAs during early embryogenesis by facilitating its helicase activity and unwinding structured 5′ untranslated regions (*45*). We show here that inhibition of DDX5 leads to a rapid dissolution of the Zb, supporting the conclusion that DDX5, at least, is a key element for the integrity of this transient compartment, potentially driving the formation of a putative amyloid-like scaffold. When Zb integrity is lost, the coupled growth of oocyte and follicle (*50*, *51*) is arrested, providing compelling evidence for the importance of Zb function in supporting normal oocyte and follicle development. Our observations are further supported by a recent study showing that conditional deletion of DDX5 in growing oocytes affects follicular growth, producing smaller ovaries specifically around 3 weeks of age, but not at earlier stages. The impact on folliculogenesis is maximal around 3 weeks of age, when antral follicle growth accelerates (*52*), in line with our observations of Zb appearance and peak abundance during this developmental window (*52*). Furthermore, loss of DDX5 in growing oocytes impairs their development and leads to female sterility (*52*).

By proposing a previously unreported role for a Balbiani body–like compartment in mammalian oocytes, our work broadens the functional repertoire of this type of structure and, as suggested in (*5*), could reveal their versatility in female germ cells.

## MATERIALS AND METHODS

### Mouse oocyte collection

All animal studies were performed in accordance with the guidelines of the European Community and were approved by the French Ministry of Agriculture (authorization number 75–1170) and by the Direction Générale de la Recherche et de l’Innovation [DGRI; Genetically Modified Organisms (GMO) agreement number DUO-5291]. Mice were housed in the animal facility on a 12-hour light/dark cycle, with an ambient temperature of 22° to 24°C and humidity of 40 to 50% and received food and water ad libitum. Mice used in this study include female C57BL/6J (Charles River Laboratories; 3 to 17 weeks old), formin-2 knockout female mice (*Fmn2^−/−^*; 8 weeks old); mice were housed in the animal facility of the Center for Interdisciplinary Research in Biology (CIRB) in Paris (authorization number D 75-05-12). For experiments on isolated oocytes, ovaries were extracted from mice as previously described (*53*) into prewarmed at 37°C M2 + bovine serum albumin (BSA; Sigma-Aldrich, A3311) medium supplemented with 1 μM milrinone (Merck, M4659) as in (*54*), which maintains growing oocytes arrested in prophase I. Ovarian follicles were punctured with surgical needles to release growing oocytes. All subsequent oocyte culture and live-imaging steps were then carried out in M2+ BSA +1 μM milrinone under oil at 37°C (Sigma-Aldrich, M8410).

### Mouse follicle collection

Follicle culture and live-imaging experiments were performed on C57BL/6J mice. All mice were female, 16 to 21 days of age, and were housed in the Marseille Institute of Developmental Biology (IBDM) mouse facility. All experiments and husbandry procedures were carried out in accordance with national and European law and were approved by the DGRI (GMO agreement number A1-1949). At the start of each experiment, fresh isolation and culture media were prepared. Isolation medium was prepared using α–minimum essential medium (α-MEM) + 25 mM Hepes (Thermo Fisher Scientific, 42360032) supplemented with penicillin-streptomycin (100 IU/ml; Gibco, 15140122), 5% fetal bovine serum (FBS; Gibco, A5669701) and 1 nM FSH [300 ng/ml; National Hormone and Peptide Programme, number NIDKK-oFSH-20 (ovine FSH)]. Culture medium was prepared without Hepes, using powdered α-MEM (Thermo Fisher Scientific, 12000014) dissolved with 0.11 g of sodium bicarbonate (final concentration, 2.2 g/liter; Sigma-Aldrich, S5761). This base medium was then supplemented with penicillin-streptomycin (100 IU/ml; Gibco, 15140122), 5% FBS (Gibco, A5669701), 1× insulin-transferrin-selenium (Gibco, 41400-045) and 1 nM FSH [300 ng/ml; National Hormone and Peptide Programme, number NIDKK-oFSH-20 (ovine FSH)] to generate the culture medium. Unless otherwise stated, culture medium additionally contained tetramethylrhodamine, methyl ester, perchlorate (TMRM; Thermo Fisher Scientific; T668; dissolved in DMSO) at a final concentration of 250 nM to visualize Zb dynamics.

To obtain groups of preantral follicles, ovaries from 16- to 21-day-old mice were collected in isolation medium. Sheets of neighboring follicles were dissected from the ovarian surface using 30-gauge needles in 35 mm–by–10 mm nontreated sterile culture dishes, each sheet containing ∼10 to 20 preantral follicles. Follicle sheets were then cultured for 24 hours in a 37°C, 5% CO_2_, 20% O_2_ incubator in a custom-built live-imaging dish. The imaging dish was constructed by removing the standing feet from the bottom of two 0.4-μm polytetrafluoroethylene (PTFE) membrane cell culture inserts (Millicell, PICM01050; 12-mm diameter) with scissors and attaching them to the bottom of each well of a glass-bottom two-well slide (ibidi, 80287) using imaging spacers (Grace Bio-Labs SecureSeal Imaging Spacers, 654002). Culture medium (0.9 ml) was added to each well, including underneath the culture insert. Follicles were transferred as small droplets onto the culture membrane using glass capillaries with an inner diameter of 0.78 mm (Harvard Apparatus, 300038), keeping them in individual droplets on the membrane. Typically, three to four groups of preantral follicles were cultured on each membrane insert. After 24 hours of culture in the incubator, 0.6 ml of the 0.9-ml culture medium was replaced, and dishes were transferred to the microscope to start imaging as quickly as possible.

### Human oocyte collection

Immature human oocytes, not arrested in meiosis II and thus not usable for patients, were collected, as approved by the local ethics committee of the Hospices Civils de Lyon (agreement number 22_5725). All patients gave informed consent. Patients undergoing assisted reproductive technologies for intracytoplasmic sperm injection (ICSI) had multifollicular ovarian stimulation. When the ovarian follicles were mature, patients underwent follicular fluid puncture to recover the cumulo-oocyte complexes (COCs). The follicular fluid was screened, and the COCs were cultured in medium [glucose in vitro fertilization (GIVF), Vitrolife, Sweden] at 37°C, 5% CO_2_, and 5% O_2_ in an incubator during 1 hour; then enzymatically (recombinant human hyaluronidase, 80 IU/ml; ICSI Cumulase, Origio); and mechanically denuded. The denuded oocytes were selected under the light microscope. Only oocytes arrested in meiosis II were used for ICSI. The other oocytes in prophase I, unsuitable for ICSI, were used in this study.

### Immunofluorescence of mouse and human oocytes

Mouse oocytes were treated with 0.4% pronase (Sigma-Aldrich, P5147) to remove the zona pellucida. Mouse and human oocytes were fixed in 4% paraformaldehyde at 30°C for 30 min. Mouse oocytes were fixed on coverslips coated with gelatin and poly-l-lysine (*3*). Mouse and human oocytes were permeabilized and preblocked for 15 min in phosphate-buffered saline (PBS) with 0.5% Triton-X (Sigma-Aldrich, 93443) and 3% BSA (Sigma-Aldrich, A2153). Oocytes were extensively washed with PBS buffer between solutions. Oocytes were incubated with primary antibodies overnight at 4°C and with secondary antibodies for 1 hour at room temperature. Coverslips were then mounted on glass microscope slides in 250-nm-thick chambers (Electron Microscopy Sciences, 70366-12) to avoid oocyte squashing, using ProLong Gold antifade reagent with 4′,6-diamidino-2-phenylindole (Thermo Fisher Scientific, P36931). Primary and secondary antibodies were diluted in PBS with 0.2% Triton-X and 3% BSA. Incubation with anti–phospho-mTOR was in PBS with 3% BSA. The following antibodies were used: rabbit anti-TOMM20 (1:800; Abcam, ab186735), mouse anti-TOMM20 (1:1200; Novus, H9804-M01), mouse anti-GM130 (1:100; BD, 610822), mouse anti-pericentrin (1:400; BD, 611814), mouse anti-DDX3 (1:800; Proteintech, 67915), mouse anti-DDX5 (1:800; Proteintech, 67025), rabbit anti-RPL7A (1:400; Sigma-Aldrich, HPA046794), rabbit anti–phospho (Ser^2448^)–mTOR (1:800; Cell Signaling Technology, 5536S), rat anti-TROMA1 [keratin 8, 1:800; Developmental Studies Hybridoma Bank(DSHB), AB531826], rabbit anti-LSM14A (1:500; Thermo Fisher Scientific, PA5-78464), and species-specific Alexa Fluor secondary antibodies (1:400, Thermo Fisher Scientific).

### Live markers and drugs

MitoTracker DeepRed (Thermo Fisher Scientific, M22426) was diluted in DMSO to make a 1 mM stock solution, used at 100 nM for 30 min; ER Tracker Red (Thermo Fisher Scientific, E34250) was diluted in DMSO at 1 mM, used at 1 μM for 20 min; SiR-tubulin (Spyrochrome, SC002) was diluted in DMSO to make 1 mM stock solution, used at 5 μM for 20 min. SYTO RNA Select (Thermo Fisher Scientific, S32703) was diluted in DMSO to make a 5 mM stock solution, used at 5 μM for 15 min; thioflavin-T (Abcam, ab120751) was diluted in DMSO to make a 100 mM stock solution, used at 10 μM for 15 min. TMRM (Thermo Fisher Scientific, T668) was diluted in DMSO at 10 mM, used at 25 nM on isolated oocytes and 250 nM on follicles throughout the time of acquisition. DND-26 LysoTracker Green (Thermo Fisher Scientific, L7526, stock solution in DMSO at 1 mM) was used at 50 nM for 20 min. 6-FAM-dC-puromycin (JenaBioscience, catalog number NU-925-6FM) was used at 50 μM for 10 min. Puromycin (Sigma-Aldrich, P7255) was diluted in water to make a stock solution at 50 mg/ml, used at 100 μg/ml for 1 hour. Cytochalasin D (Sigma-Aldrich, C8273) was diluted in DMSO at 10 mg/ml, used at 1 μg/ml for 30 min. Nocodazole (Sigma-Aldrich, M1404) was diluted in DMSO at 10 mM, used on oocytes at 1 μM for 30 min. RK-33 (MedChemExpress, HY-100455) was diluted in DMSO at 5 mM, used on oocytes at 10 μM throughout the time of acquisition. Supinoxin (Tebubio, T16961) was diluted in DMSO at 10 mM, used on oocytes at 200 nM throughout the time of acquisition.

For isolated oocytes, culture medium containing live markers or drugs at their working concentration was freshly prepared and prewarmed at 37°C. For follicle live imaging, DMSO or drug treatments were added at 10× the desired final concentration in 99 μl of culture medium to the 900 μl in the well of the imaging dish immediately before acquisitions began. All stock solutions and aliquots were stored at −20°C.

Where applicable, each experiment in this study contained internal controls to ensure that variability between experimental replicates did not bias the outcome of the experiments.

For follicle live imaging, most experiments used follicles collected from multiple mice pooled together before random assignment to control and treatment groups. Control (DMSO-treated) follicles were always cultured and handled alongside drug-treated follicles. Follicles belonging to each treatment condition were processed and analyzed blindly to avoid bias.

### Microinjection

Oocytes were microinjected with complementary RNAs (cRNAs) using an Eppendorf Femtojet microinjector. Oocytes were kept in prophase I between 2 and 4 hours to allow expression of fusion proteins. All live culture and imaging were carried out under oil at 37°C. We used the following constructs: mCherry-PLK4 (*55*), EB3-GFP (*56*), and DDX3X-GFP (this study). pRN3-Ddx3x-GFP was obtained by subcloning *Ddx3x* ORF clone (CliniSciences, MG226484) into the Eco RI–Sal I sites of a pRN3-GFP vector (GenScript). In vitro synthesis of capped cRNAs was performed as previously described (*57*). cRNAs were centrifuged at 4°C for 45 min at 13,000 rpm before microinjection.

### Fixed and live imaging

Spinning-disk images were acquired at 37°C using a Plan-APO 40×/1.25 numerical aperture (NA) objective on a Leica DMI6000B microscope enclosed in a thermostatic chamber (Life Imaging Service) equipped with a Retiga 3 CCD camera (QImaging, Burnaby) coupled to a Sutter filter wheel (Roper Scientific) and a Yokogawa CSU-X1-M1 spinning-disk. Metamorph software (Molecular Devices) was used to collect mouse oocytes data. To follow EB3-GFP labeling of microtubules, mouse oocytes were imaged every 500 ms with the stream acquisition mode of Metamorph upon 491-nm excitation. All other live and fixed mouse oocytes samples were acquired with 3-μm *z*-step over a 27-μm-thick slice. Time-lapse images of oocytes after nocodazole washout were acquired every 20 or 30 min. Time-lapse images of oocytes after 6-FAM-dC-puromycin treatment were acquired every 30 s with 0.5-μm *z*-step over a 4.5-μm-thick slice. Exposition was 500 ms in all channels for all acquisitions.

Human oocytes were placed in a FluoroDish (World Precision Instruments, number FD35–100) in small drops of PBS with 3% BSA covered with mineral oil (Ovoil, Vitrolife, Sweden). Imaging was performed with a spinning-disk microscope (Olympus IX 83 Inverted microscope, equipped with a Yokagawa CSU-X1 spinning-disk). Acquisitions were taken using transillumination and fluorescence at 40× with 20 *z*-steps spaced of 4 μm. Metamorph software (Universal Imaging) was used to collect data, and ImageJ (National Institutes of Health) was used to process data.

For follicle live imaging, the imaging chamber was supplied with 5.5% CO_2_ and heated such that the imaging medium measured at 37.5°C. All microscopy was performed using ZEN Blue 3.10 and ZEN Black 2.3 (Zeiss). Images were acquired using LSM 880 confocal laser scanning microscopes or a Celldiscoverer 7 (CD7) with LSM 900 (Zeiss), each equipped with an environmental incubator box. On the LSM 880, a LD LCI Plan-Apochromat 25×/0.8 Imm Korr DIC M27 objective was used on the water-immersion setting in combination with Immersol W2010 immersion oil. On the CD7, imaging was performed with the autoimmersion objective (50×) and an Optovar 0.5× (Zeiss). Control and drug-treated groups were imaged on the same microscope. Care was taken to avoid phototoxicity and photobleaching.

### Scanning electron microscopy

For each experimental condition, groups of about 10 oocytes per stage were adhered on a round glass coverslip (12-mm diameter, 150-μm thickness) previously coated with gelatin and polylysine with etched landmarks. Coverslips were prepared in a 24-well plate, at room temperature (except when mentioned). Samples were fixed 1 hour with 2% glutaraldehyde and 0.04% ruthenium red in 0.1 M sodium cacodylate buffer (pH 7.4), washed with the same buffer, postfixed 1 hour with 1% osmium tetroxide and 1.5% potassium ferrocyanide in 0.1 M cacodylate buffer at 4°C, and washed with ultrapure water. Samples were dehydrated at 4°C with ethanol [50, 70, 95, and (3×) 100%; 5 to 15 min each step] and infiltrated during 24 hours with resin [25, 50, and (2×) 100%; Agar 100 kit, last step in vacuum]. Coverslips were adhered on glass slides with a drop of resin and covered with upside-down gelatin capsules filled with resin and were polymerized 48 hours at 60°C. Capsules were then detached with the heat of a lighter under the glass slides (*58*). Blocks were cut with an ultramicrotome (Ultracut UCT, Leica microsystems) and a diamond knife (Ultra 35°, Diatome) until reaching the plane of maximum diameter of the oocyte. Thin sections (80 nm) were deposited on 5-mm square silicon wafers and were contrasted 15 min with 2.5% uranyl acetate. Wafers were stuck on aluminum stubs with silver paint. Sections were observed with a field emission scanning electron microscope (Gemini 500, Zeiss), operating in high vacuum, at 1.5 kV, with the high current mode and a 60-μm aperture diameter (corresponding to a specimen current of 411 pA) and a working distance around 2 mm. An in-column detector was used to collect backscattered electrons (filtering grid at 600 V). LookUp Table was inverted to obtain electron microscope-like images. Automated acquisitions were performed using Atlas 5 (Fibics), with a pixel dwell time of 51.2 μs, a pixel size of 3 nm, an image definition of 7000 × 7000 pixels, and an overlap of 15% between images. Full maps of oocytes (six oocytes per stage) were obtained after stitching the image mosaics.

### RNA-seq and differential gene expression analysis

A total of five C57BL/6J female mice (Charles River laboratories) aged 4 weeks were used for the experiments. Oocytes at the cluster stage were isolated in culture medium and transferred in PBS. Oocytes were either lysed to collect isolated Zbs or collected whole for control samples, in triplicate. Each sample contained either 10 isolated Zbs or 8 whole oocytes at the cluster stage. Individual samples were collected in 2 μl of PBS, then resuspended in lysis buffer supplemented with 1% of β-mercaptoethanol, frozen in liquid nitrogen, and conserved at −80°C overnight. Total RNA was extracted using the RNAqueous Micro Total RNA Isolation Kit (Thermo Fisher Scientific, AM1931). After unfreezing, RNA extraction was carried out following the manufacturer’s instructions. RNA was eluted twice in 10 μl of elution buffer. Samples were then treated with deoxyribonuclease I and stored at −80°C. Total RNA (9.5 μl) was amplified and converted to cDNA using a SMART-Seq mRNA kit (Takara). Afterward, an average of 500 pg of amplified cDNA was used to prepare library following Nextera XT DNA kit (Illumina). Libraries were multiplexed by 6 on a P2 flow cells. A 118–base pair read sequencing was performed on a NextSeq 2000 device (Illumina). A mean of 87 ± 7 million passing Illumina quality filter reads was obtained for each of the six samples.

The analyses were performed using the Eoulsan pipeline (*59*), including read filtering, mapping, alignment filtering, read quantification, normalization, and differential analysis. Before mapping, poly N read tails were trimmed, reads ≤40 bases were removed, and reads with quality mean ≤30 were discarded. Reads were then aligned against the *Mus musculus* genome from Ensembl version 105 using STAR (version 2.7.8a) (*60*). Alignments from reads matching more than once on the reference genome were removed using Java version of SAMtools (*61*). To compute gene expression, *M. musculus* genome annotation from Ensembl version 105 database was used. All overlapping regions between alignments and referenced exons were counted and aggregated by genes using HTSeq-count 0.5.3 (*62*). The sample counts were normalized using DESeq2 1.8.1 (*63*). Statistical treatments and differential analyses were also performed using DESeq2 1.8.1. DESeq2 uses a negative binomial model, normalizing with the median of ratios method. Wald test is used to identify differentially expressed genes, and false discovery rate (FDR) with Benjamini-Hochberg method is applied to obtain the adjusted *P* value corrected for multiple comparisons. The RNA-seq data are available on the following link: www.ebi.ac.uk/biostudies/ArrayExpress/studies/E-MTAB-16643?key=d23c4db4-9b02-4cfa-889f-c2708c8a8d51.

### Mass spectrometry and enrichment analysis

A total of 14 C57BL/6J female mice (Charles River laboratories) aged 4 weeks were used for the experiments. Prophase I–arrested oocytes were collected in M2 + polyvinyl alcohol (PVA) medium supplemented with 1 μM milrinone. Clean glass pipettes with a diameter of about 20 μm were used to disrupt cluster stage oocytes and isolate the Zb. Between 50 and 60 isolated Zbs or 50 whole oocytes at the cluster stage were collected in 1 μl of medium, transferred into 15 μl of 6 M guanidinium chloride, and stored at −80°C until processing. Samples were processed for mass spectrometry and analyzed as described (*64*). Results in table S2: Sample S1 contained all collected isolated Zbs, whereas samples S2 and S3 contained 10 and 20%, respectively, of the whole cluster stage oocyte sample. Raw data were filtered to remove contaminants (“CON” in Accession column) and to keep only proteins identified with a high confidence (Protein FDR Confidence column). Because the total protein content was different in each sample, the relative abundance of each protein was calculated as a percentage in the three samples: (Abundance value of a protein in a sample S/total protein content of S)*100, where the total protein content corresponds to the sum of abundance values of all proteins found in the sample. The ratio between the relative abundance from samples 1 and 3 was then calculated. To identify the proteins enriched in the isolated Zb, a filter was applied to the “Found in Sample S1” column to remove the “Not Found” occurrence; proteins were then ordered in decreasing value of ratio between relative abundances of sample 1 and sample 3. Proteins found in the isolated Zb whose ratio in relative abundance compared with those in sample 3 was >1 were considered enriched in the isolated Zb. To check for PrLDs, protein sequences were obtained from UniProt (www.uniprot.org/id-mapping) and uploaded on PLAAC website [Prion-like Amino Acid Composition (*65*), http://plaac.wi.mit.edu/details].

### Gene enrichment analyses

For the GSEA, normalized count matrices were used from RNA-seq data to generate expression dataset and phenotype labels files as from GSEA 4.4.0 software guidelines (*66*). GSEA was conducted using the “m5.go.cc.v2025.1.Mm.symbols.gmt” and “m5.go.mf.v2025.1.Mm.symbols.gmt” Gene Ontology databases for pathway analysis (*67*, *68*). Mouse Ensembl Gene ID chip was used to collapse dataset to symbols before analysis, with permutation type “gene_set.” Weighted enrichment statistics were used with “Signal2Noise” ranking gene metric. Gene sets larger than 500 and smaller than 15 were excluded. Dot plot was generated with R studio for the top 20 enriched pathways using the clusterProfiler, enrichplot, dplyr, and ggplot2 packages (*69*, *70*). Pathways were ranked according to the normalized enrichment score, which accounts for differences in gene set size and variability between gene sets and the expression dataset. Only Gene Ontology pathways identified as significantly enriched by GSEA were included. *P* values were adjusted using the Benjamini-Hochberg FDR method. “Count” indicates the number of genes in each pathway on the *y* axis.

Data obtained from mass spectrometry were analyzed with ShinyGO V0.81 (*71*) on the total list of proteins identified in the isolated cluster and using the list of proteins identified in the whole cluster stage oocyte as background. The FDR is calculated from the nominal *P* values obtained using the hypergeometric test. Fold enrichment is defined as the proportion of genes in the input list that belong to a given pathway, divided by the corresponding proportion in the background set. To identify possible PrLDs, primary sequences of proteins identified by mass spectrometry were analyzed using PLAAC (*65*).The same software was used to generate protein sequence representation in fig. S5A.

### Fluorescence recovery after photobleaching

Oocytes expressing DDX3X-GFP were used to perform fluorescence recovery after photobleaching (FRAP) assays. FRAP acquisitions were performed on a Plan-APO 60×/1.4 NA objective on a spinning disk confocal microscope (Nikon Eclipse Ti, Nikon) equipped with a spinning-disk head (X1, Yokogawa), an Evolve EMCCD camera (Photometrics), and a thermostatic chamber (37°C, 5% CO_2_). The iLAS 2 module (Gataca System) was used to control the FRAP laser. Excitation was carried out on a fixed circular region of ∼10 μm of diameter and three equally sized regions in the surrounding cytoplasm. High-intensity laser pulses (100% of 488-nm laser power, 20 pulses for a total of 0.48 ms of stimulation) were applied to photobleach fluorescence in these regions. Fluorescence recovery was then recorded in time-lapse mode with an interval of 500 ms over a total duration of 300 s. A preacquisition phase of 10 time points was used to establish a baseline fluorescence level. FRAP image sequences were realigned with Fiji StackReg plugin, and regions of interest (ROIs) were selected for fluorescence recovery analysis. A spreadsheet was generated with the fluorescent intensity value of bleached regions in the cluster or cytoplasm at every time point, as well as of the whole cell and background. The spreadsheet was then uploaded on EasyFRAP-web (*72*). Recovery was measured as fluorescence intensity of photobleached regions corrected for background and normalized by the region’s prebleach and postbleach intensities. Data were plotted on GraphPad Prism 7 to obtain graph in fig. S5E.

### Nascent protein synthesis using the OPP kit

The Click-iT Plus OPP Alexa Fluor 647 Protein Synthesis Assay Kit (Thermo Fisher Scientific, C10458) was used for the detection of protein synthesis. Prophase I oocytes at the cluster stage were treated or not with puromycin (100 μg/ml) for 1 hour before incubation with the puromycin analog *O*-propargyl-puromycin (OPP) at 10 μM for 30 min and before their fixation. OPP detection was carried out following the manufacturer’s instructions.

### Nascent protein synthesis using 6-FAM-dC-puromycin

Cluster stage oocytes were processed individually. Each oocyte was preincubated in 100 nM MitoTracker DeepRed for 20 min and then incubated in 50 μM 6-FAM-dC-puromycin with 100 nM MitoTracker DeepRed for 10 min. Oocytes were transferred directly to glass-bottom eight-well slide (ibidi, 80807) with 0.4 ml of culture medium added in each well before imaging.

### Oocyte segmentation and feature extraction using Oocytor

The pipeline based on Oocytor (*73*) was applied to transmitted-light images acquired for dry-mass measurement. As a first step, Oocytor detects the oocyte cortex and zona pellucida contours from transmitted-light images based on neural networks [with a U-Net architecture; (*74*)]. A model previously trained on images from both mouse and human oocyte was used (accessible here: https://github.com/gletort/Oocytor/blob/main/models/cortex/general.zip for cortex segmentation and https://github.com/gletort/Oocytor/tree/main/models/zp for zona pellucida segmentation). Dry-mass raw images contain the refractive index information in multiple angles and present a checkboard-like pattern at the pixels level. At the full image level, the main components of the oocyte are, nevertheless, visible so we were able to run Oocytor directly on these images without having to retrain the networks on these specific types of images. However, as the neural networks were not fine-tuned, all the results were manually checked, and about one-third were badly segmented. We preprocessed the imaged for which the segmentation failed by readjusting the crop, converting the image to 8-bit, smoothing the pixel values (“Smooth” function of Fiji), shifting the pixel value distribution to a mean of 100, and, sometimes, with contrast enhancing (“Enhance Contrast” function of Fiji). These preprocessing steps allowed making the images more similar to the training dataset. Oocytor was run again on the reprocessed images and gave better segmentation results. In a second step, we extracted morphological values describing the oocyte. Oocytor proposes features based either on the segmentation contour or on the intensity distribution. Here, as the pixel values reflect different refractive indexes and the images were not all preprocessed in the same way, we did not analyze the intensity-based measures.

### Dry-mass measurement

A commercial PHASICS SID4-sC8 wave front sensor, recording phase images with up to 720 × 720 pixels (pixel size of 19.5 μm), was used. The dry mass of oocytes was measured according to (*75*). To image the oocytes, the aperture diaphragm was closed to the smallest size to ensure that the sample was illuminated by a highly coherent and plane wave. A reference wave front was acquired close to the oocyte to measure. The Oocytor plugin (*73*) was used to segment oocytes from the interferograms acquired with the wave front sensor. A fourth-order polynomial fit of the optical path difference (OPD) values was made and subtracted to the whole-phase images, including the oocyte pixels. The resulting OPD values inside the oocyte ROI determined by the Oocytor plugin were summed to extract the dry mass. The oocyte mean diameter was deduced from the ROI area, assuming the oocyte projection in the equatorial plane as a disk.

### Software used for drawings

Adobe Illustrator was used for drawings in Figs. 1H, 3A, and 4 (A and C). Drawing for Fig. 1G was created in BioRender (agreement number UW29WV8CZY to C.T., 2026).

### Image analysis and quantifications

All images were analyzed on Fiji [version 2.1.0/1.53c; (*76*)].

Quantification of oocytes and Zb surface in Fig. 1B was obtained from live oocytes labeled with MitoTracker DeepRed.

Quantification of follicles, oocytes, and Zb surface in Fig. 6 (B and D to I) and fig. S3 (B, C, F, and G) was obtained from live follicles labeled with TMRM.

For follicle live imaging, sample size was determined by the maximum number of follicles that fit on the imaging membrane. This was typically three to four groups of 10 to 20 follicles each for control and drug inhibition conditions. No statistical methods were used to predetermine sample sizes. Follicles were not used for live imaging if they were deemed unhealthy on the basis of both follicle and oocyte morphology. To calculate follicle and oocyte areas, images of individual oocyte-containing follicles were imported into Fiji/ImageJ. ROIs were hand-drawn on single-plane transmitted-light images, and area measurements were extracted for all time points.

For the quantification of fluorescence intensity ratio between Zb and cytoplasm in Fig. 5 (C and D) and fig. S5I, the ROIs outlining the Zb, the nucleus, and the whole oocyte were defined for each oocyte on a single-plane image based on the signal from MitoTracker, TOMM20, GM130, or autofluorescence of organelles at 488 nm. Cytoplasmic signal was obtained on the whole-oocyte ROI excluding signal from the Zb and the nucleus, using the “XOR” function in Fiji. Signal intensity was measured after background removal on Fiji. The ratio of the signal intensity in the Zb versus cytoplasm was calculated on Excel and plotted on GraphPad Prism 7.

Phospho-mTOR intensity in Fig. 5G corresponds to the mean gray value of the maximal intensity z projection for the whole oocyte. Quantifications of zona pellucida width in fig. S1B were obtained with Oocytor (*73*). Quantification of the number of oocytes with or without the cluster at different mouse ages from fig. S1C was performed on live oocytes stained with MitoTracker and/or SYTO RNA Select. Quantification of Zbs dissolved by nocodazole treatment in fig. S2F was estimated on data from assays from fig. S2 (H and I) (for 30 min) and Fig. 5 (E and F) (for 2 hours). Quantification of Zb size after nocodazole treatment from fig. S2G was performed on oocytes treated for 2 hours as in Fig. 5 (F and G).

### Statistical analysis

All graphs and statistical analyses were generated using MS Excel (version 16.78) and GraphPad Prism 7, 9, or 10. SD was used as *y*-axis error bars for bar charts and curves plotted from the mean value of the data. Statistical significance based on one way analysis of variance (ANOVA) followed by Tukey’s post hoc test, unpaired *t* test with Welch’s correction, or Mann-Whitney test was calculated in GraphPad Prism 7. Linear regression analysis was performed in GraphPad Prism 9. Each experimental condition reported included at least two independent replicates. Biological replicates are indicated in the manuscript alongside the data figures, in the figure legends, and/or in Materials and Methods. In the figures, significance is designated as follows: *P < 0.05, **P < 0.01, ***P < 0.001, and ****P < 0.0001. Nonsignificant values are indicated as n.s.
