# Supplementary material for: A transient RNP compartment boosts translation and growth of mammalian oocytes
Source: Sci Adv. 2026 Aug 28;12(35):eaej5359. doi: 10.1126/sciadv.aej5359 (PMC13524057; doi:10.1126/sciadv.aej5359)
Supplement: Supplementary file 1 [file sciadv.aej5359_sm.pdf]

Science Advances
AAAS

Supplementary Materials for

A transient RNP compartment boosts translation and growth of mammalian oocytes

Noemi Zollo *et al.*

Corresponding author: Christopher Thomas, christopher.thomas@univ-amu.fr;
Maria Almonacid, maria-elsa.almonacid@college-de-france.fr;
Marie-Hélène Verlhac, marie-helene.verlhac@college-de-france.fr

Sci. Adv. **12**, eaej5359 (2026)
DOI: 10.1126/sciadv.aej5359

The PDF file includes:

Figs. S1 to S6
Legends for tables S1 and S2
Legend for movie S1

Other Supplementary Material for this manuscript includes the following:

Tables S1 and S2
Movie S1

Supplementary Material Legends:

Fig. S1. The cluster is found at a specific stage in mammalian oocyte growth. (A) Transmitted light images of oocyte at the Cluster (left), Intermediary (middle) and Fully grown (right) stages. **(B)** Quantification of Zona Pellucida (ZP) thickness for oocytes at the Cluster, Intermediary or Fully grown stage, performed with the AI tool Oocytor. N=53 oocytes for Cluster stage, N=29 oocytes for Intermediary stage, N=41 oocytes for Fully grown stage. **(C)** Proportion of oocytes at Cluster vs Intermediary or Fully grown stages at different mouse ages. N=78 oocytes, 2 experiments for PN12; N=333 oocytes, 4 experiments for PN16; N=555 oocytes, 4 experiments for 3 weeks; N=415 oocytes, 6 experiments for 4 weeks; N=197 oocytes, 4 experiments for 5-10 weeks; 162 oocytes, 5 experiments for >10 weeks. **(D)** Late non-fully grown human oocyte (left) presenting an accumulation of mitochondria adjacent to its nucleus; the chromatin architecture corresponds to a stage before the fully-grown one. Fully grown human oocyte (right) displaying an homogeneous distribution of mitochondria. Mitochondria stained with TOMM-20 in pink and Chromosomes stained with DAPI in green. Scale bar is 10 µm. N=27 human oocytes.

Fig. S2. The integrity of the cluster is linked to microtubules. (A) Cluster stage oocyte treated with Nocodazole (Noco, left), Cytochalasin D (Cyto D, middle) or collected from *Fmn2*$^{-/-}$ mice (right), stained with SYTO RNA Select. **(B)** Cluster stage oocyte stained for mitochondria (Tomm20, left) and Pericentrin (middle) to visualize the MTOC. The right panel shows the merge of Tomm20 (pink) and Pericentin (blue) staining. **(C)** Cluster stage oocyte stained with Mito Tr (left) and co-expressing EB3-GFP (EB3, middle left) to visualize microtubules growing (+) ends and mcherry-Plk4 (Plk4, middle right) to visualize MTOC. The right panel shows the merge of Mito Tr (pink), EB3 (blue) and Plk4 (yellow) staining. **(D)** Temporal color-coded projection of a stream movie on EB3-GFP (left) and its zoom (dashed white square, right); acquisition every 500 ms for 50 s. **(E)** Representative images of a Cluster stage oocyte stained with SYTO RNA (left) and SiR Tubulin (right). **(F)** Percentage of oocytes at the Cluster stage whose Zb dissolved completely or not after 30 min or 2 h treatment with 1 µM Nocodazole (Noco); N= 70 oocytes for 30 min treatment, 6 independent experiments; N=45 oocytes for 2 h, 5 independent experiments. **(G)** Quantification of cluster surface in oocytes at the Cluster stage treated with 1 µM Noco or its control DMSO for 2 h from (F); N=39 oocytes for DMSO, N=45 oocytes for Noco. **(H)** Representative images of a Cluster stage oocyte stained with Mito Tr, treated 30 min with 1 µM Nocodazole, then washed out and observed at different time points. **(I)** Quantification of cluster reformation after Noco washout as in (H). Only oocytes with completely dissolved cluster were selected for quantification after Noco washout. N=15 oocytes for Noco; N=9 oocytes for DMSO. 2 independent experiments. Data are shown as mean ± SD. P values were calculated using one-way ANOVA with Tukey's post hoc test. Scale bars 10 µm. ns = not significant; * = p<0.05; ** = p<0.01; *** = p<0.001; **** = p<0.0001.

Fig. S3. Live imaging of growing Zbs within their physiological follicular environment. (A) Transmitted light image of a mechanically isolated group of follicles (left), stained with TMRM (center); zoomed insets on the right show follicles containing Cluster stage oocytes with a Zb. Scale bars are 50 µm. **(B)** Quantification of Zb over oocyte diameter, measured at their maximal cross section in Cluster stage oocytes within follicles. N=53 follicles for both Zb and oocyte measurements; 4 independent experiments. **(C)** Proportion of Cluster stage oocyte with or without a Zb found in follicles of different sizes obtained as from Fig. 1G. The percentage of follicles presenting a Zollo body is indicated for each size range. N=13 follicles

for size 50-80 μm (early secondary); N=91 follicles for size 80-170 μm (secondary); N=13 follicles for size 170-230 μm (early antral); N=6 follicles for size >230 μm (antral); 6 independent experiments. **(D)** Representative images of a Cluster stage oocyte within its follicle stained with TMRM, observed at different time points. Pink arrowheads highlight the formation of the *antrum*. Scale bars are 10 μm. **(E)** Zoomed inset of the Zb from (D) at different timepoints; yellow arrowheads indicate mitochondria as they are incorporated in the Zb. Representative image pairs were acquired at 15 minutes intervals. Scale bars are 10 μm. **(F)** Quantification of size changes in three Zbs in the follicular environment, considering the last 20 hours before the Zb reached its maximal size. **(G)** Quantification of Cluster stage oocyte over follicle surface, measured at their maximal cross section. N=53 for both oocyte and follicle measurements; 4 independent experiments.

Fig. S4. Molecular Function Gene Set Enrichment Analysis from the RNA seq of the isolated Zb. (A) Cluster stage oocyte stained with Mito Tracker (Mito Tr, left) and Lyso Tracker (Lyso Tr, middle). The right panel shows the merge of Mito Tr (pink) and Lyso Tr (blue) staining. Scale bars are 10 μm. **(B)** Enriched GO pathways in the category Molecular Function (from up-regulated genes presented in Fig 3C and Table S1).

Fig. S5. The Zb contains prion-like proteins but does not contain the canonical RNP marker Lsm14a. (A) PLAAC algorithm results predicting prion-like domains in proteins from the Zb from the mass spectrometry data (Fig 4A and Table S2). Pink shading indicates predicted prion-like sequences. **(B)** Cluster stage oocyte stained for Tomm20 (left) and Ddx3x (middle). The right panel shows the merge of Tomm20 (pink) and Ddx3x (blue) staining. **(C)** Cluster stage oocyte stained for Tomm20 (left) and Ddx3x (middle). The right panel shows the merge of Tomm20 (pink) and Ddx5 (blue) staining. **(D)** FRAP experiment performed on Cluster stage oocyte expressing Ddx3x-GFP. Dotted pink and black circles are regions of interest bleached respectively inside the Zb and inside the surrounding cytoplasm. **(E)** Quantification of the FRAP experiment in (D). The pink and black recovery curves correspond to regions in the Zb and in the cytoplasm, respectively. N= 21 oocytes; 3 independent experiments. **(F)** Staining indicating the absence of Lsm14a (middle) inside the Zb, identified by Golgi (GM130, left). The right panel shows the merge of Golgi (pink) and Lsm14a (blue) staining. **(G)** Cluster stage oocyte stained to visualize the ribosomal subunit Rpl7 (middle); the Zb is visualized via autofluorescence of its organelles at 488 nm (organelles, left). The right panel shows the merge of mitochondria (pink) and Rpl7 (blue) staining. **(H)** Staining indicating enrichment of Keratin 8 (Krt8, middle) in the Zb, identified by mitochondria (Tomm20, left). The right panel shows the merge of mitochondria (pink) and Krt8 (blue) staining. **(I)** Quantification of Ddx3, Ddx5, Lsm14a, Rpl7 and Krt8 signal intensities in the Zb compared to the cytoplasm from (B), (C), (F), (G) and (H). N= 30 oocytes for Ddx3x, 4 independent experiments; N=20 oocytes for Ddx5, 3 independent experiments; N=20 oocytes for Lsm14a, 3 independent experiments; N= 42 oocytes for Rpl7, 3 independent experiments; N=21 oocytes for Krt8, 3 independent experiments. Data are shown as mean ± SD. P values were calculated using unpaired two-tailed Student's t test. Scale bars are 10 μm. **** = $p<0.0001$.

Fig. S6. Treatment with 5h puromycin impacts the dry mass of oocytes at the Cluster stage. (A) Oocyte diameter at Cluster, Intermediary and Fully grown stages measured with quantitative phase imaging after 5h puromycin treatment. N= 48 oocytes for Cluster ctrl, N= 37 oocytes for Intermediary ctrl, N= 78 oocytes for Fully grown ctrl; N= 40 oocytes for Cluster puromycin, N= 47 for Intermediary puromycin, N= 72 oocytes for Fully grown puromycin; 3 independent experiments. **(B)** Oocyte dry mass at Cluster, Intermediary and Fully grown stages measured with quantitative phase imaging after 5h puromycin treatment. N= 48 oocytes for

Cluster ctrl, N= 37 oocytes for Intermediary ctrl, N= 78 oocytes for Fully grown ctrl; N= 40 oocytes for Cluster puromycin, N= 47 oocytes for Intermediary puromycin, N= 72 oocytes for Fully grown puromycin; 3 independent experiments. **(C)** Oocyte protein density at Cluster, Intermediary and Fully grown stages estimated from measurements in (A) and (B). N= 48 oocytes for Cluster ctrl, N= 37 oocytes for Intermediary ctrl, N= 78 oocytes for Fully grown ctrl; N= 40 oocytes for Cluster puromycin, N= 47 oocytes for Intermediary puromycin, N= 72 oocytes for Fully grown puromycin; 3 independent experiments. **(D)** Oocyte diameter at Cluster, Intermediary and Fully grown stages measured with quantitative phase imaging after 5h Nocodazole treatment. N= 42 oocytes for Cluster DMSO, N= 29 oocytes for Intermediary DMSO, N= 36 oocytes for Fully grown DMSO; N= 48 oocytes for Cluster Nocodazole, N= 30 oocytes for Intermediary Nocodazole, N= 36 oocytes for Fully grown Nocodazole; 3 independent experiments. **(E)** Oocyte dry mass at Cluster, Intermediary and Fully grown stages measured with quantitative phase imaging after 5h Nocodazole treatment. N= 42 oocytes for Cluster DMSO, N= 29 oocytes for Intermediary DMSO, N= 36 oocytes for Fully grown DMSO; N= 48 oocytes for Cluster Nocodazole, N= 30 oocytes for Intermediary Nocodazole, N= 36 oocytes for Fully grown Nocodazole; 3 independent experiments. **(F)** Oocyte protein density at Cluster, Intermediary and Fully grown stages estimated from measurements in (D) and (E). N= 42 oocytes for Cluster DMSO, N= 29 oocytes for Intermediary DMSO, N= 36 oocytes for Fully grown DMSO; N= 48 oocytes for Cluster Nocodazole, N= 30 oocytes for Intermediary Nocodazole, N= 36 oocytes for Fully grown Nocodazole; 3 independent experiments. Data are shown as mean ± SD. P values were calculated using Mann-Whitney test. ns = not significant; * = $p<0.05$.

Table S1. RNA sequencing results: isolated Zb vs Cluster stage oocytes. First sheet: summary of file content. Second sheet: raw sample count. Third sheet: differential gene expression obtained from DEseq analysis. Fourth sheet (pink): list of downregulated RNAs with a log2 FC < -1 and p value < 0.05. Fifth sheet (green): list of upregulated RNAs with a log2 FC > 1 and p value < 0.05.
Table S1 refers to Fig. 3.

Table S2. Mass Spectrometry analysis on isolated Zb vs Cluster stage oocytes. First sheet: Samples description and methodology. About 50-60 isolated Zbs were processed (sample S1=100% of total submitted sample), with 50 whole oocytes at the Cluster stage as control (sample S2=10% of total submitted sample; sample S3=20% of total submitted sample). Second sheet: raw data as provided by the platform. Third sheet (yellow): result of the enrichment analysis as described in Methods. Fourth sheet (pink): common genes identified in the isolated Zollo body between the lists obtained by RNA sequencing (Table S1) and mass spectrometry.
Table S2 refers to Fig. 4.

Movie S1. Zollo body in motion. 2.5 days of dynamic remodeling of the Zollo body in a follicle-enclosed oocyte. Labelling with TMRM; scale bar 50 μm.

Fig. S1

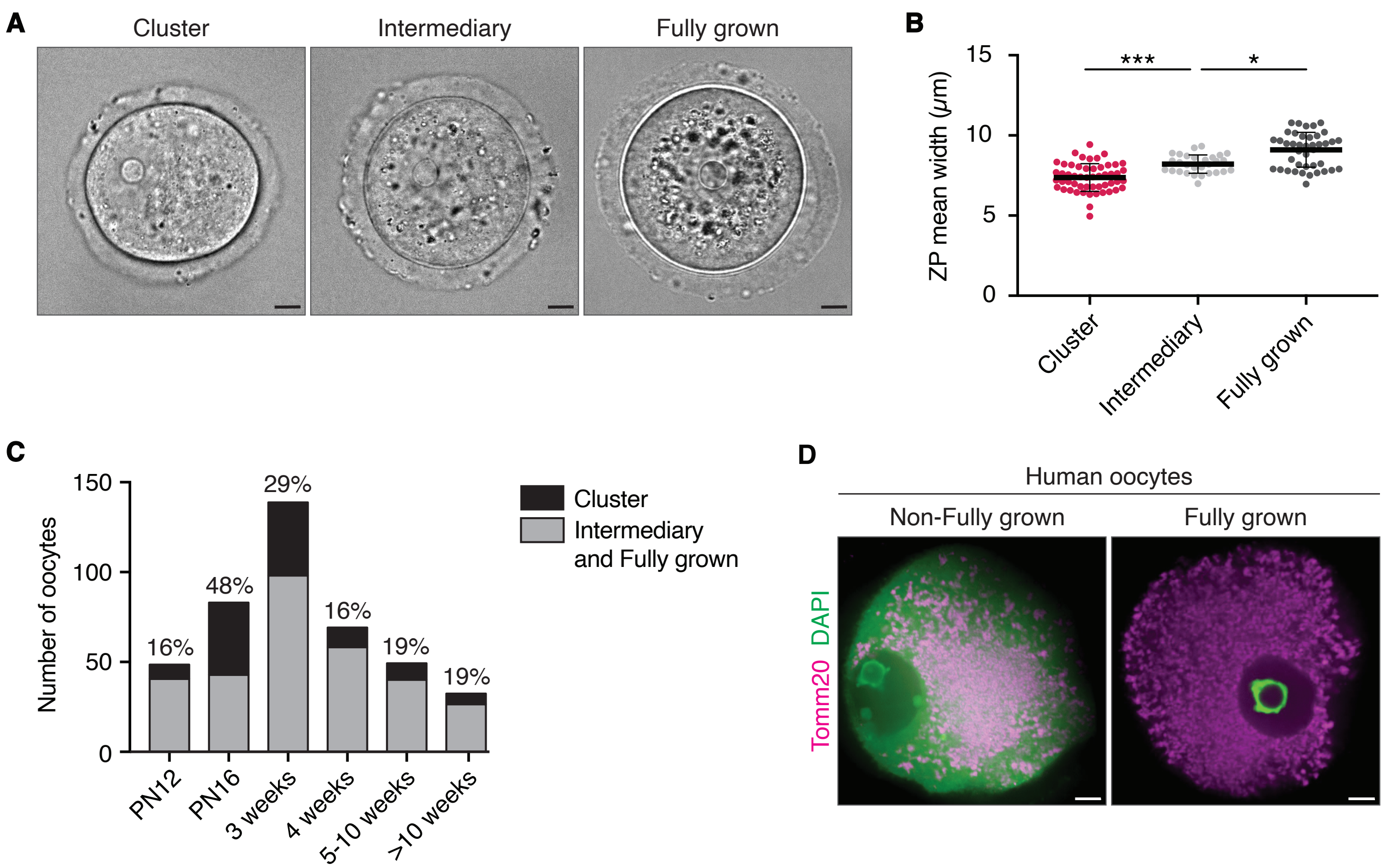
A
Cluster
Intermediary
Fully grown
B
ZP mean width (μm)
15
10
5
0
***
*
Cluster
Intermediary
Fully grown
C
Number of oocytes
150
100
50
0
16%
48%
29%
16%
19%
19%
PN12
PN16
3 weeks
4 weeks
5-10 weeks
>10 weeks
Cluster
Intermediary
and Fully grown
D
Human oocytes
Non-Fully grown
Fully grown
Tomm20 DAPI

Fig. S2

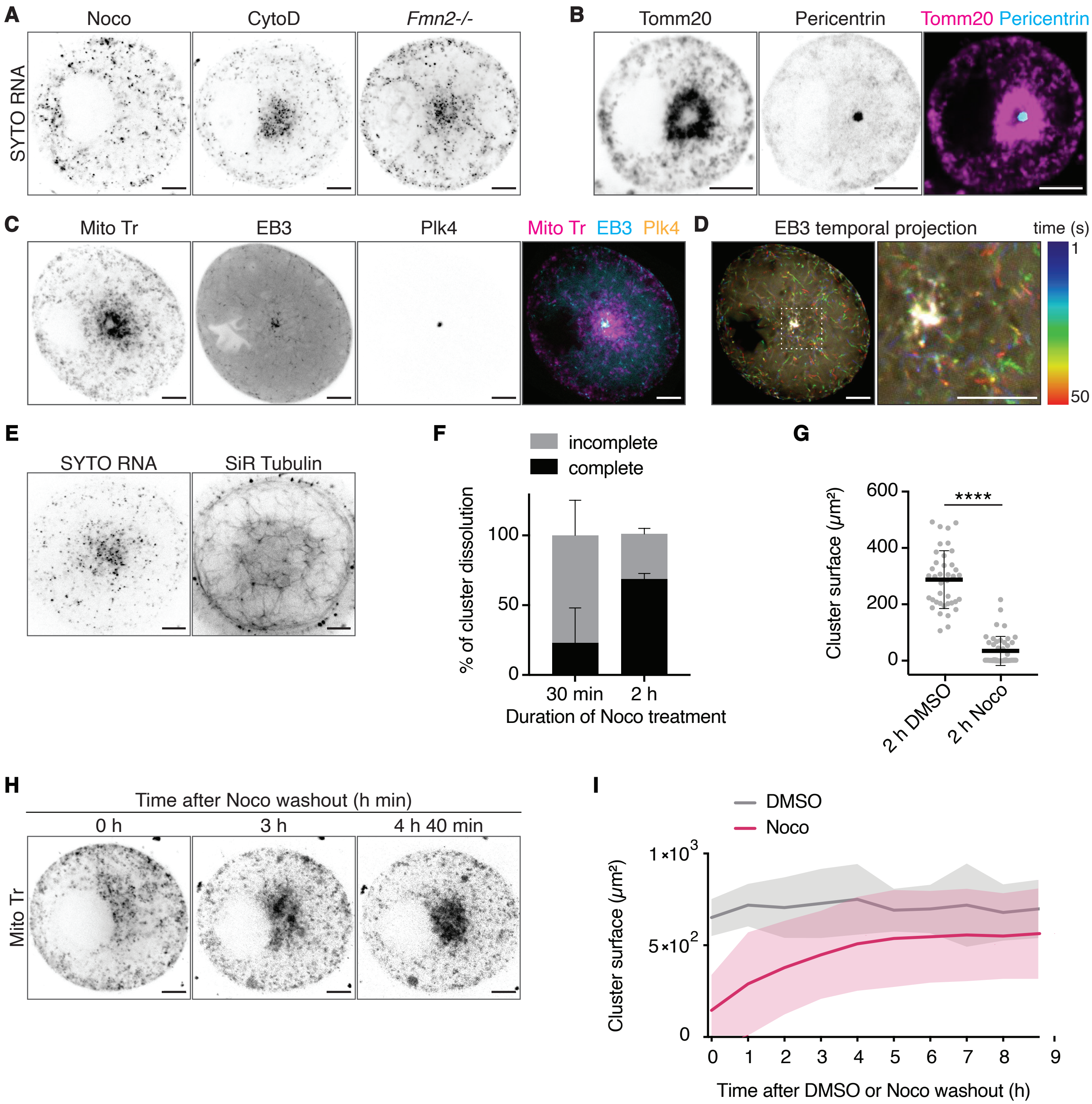

A
Noco
CytoD
Fmn2-/-
SYTO RNA
B
Tomm20
Pericentrin
Tomm20 Pericentrin
C
Mito Tr
EB3
Plk4
Mito Tr EB3 Plk4
D
EB3 temporal projection
time (s)
1
50
E
SYTO RNA
SiR Tubulin
F
incomplete
complete
% of cluster dissolution
100
50
0
30 min
2 h
Duration of Noco treatment
G
Cluster surface (µm²)
600
400
200
0
****
2 h DMSO
2 h Noco
H
Time after Noco washout (h min)
0 h
3 h
4 h 40 min
Mito Tr
I
DMSO
Noco
Cluster surface (µm²)
1 ×10³
5 ×10²
0
0
1
2
3
4
5
6
7
8
9
Time after DMSO or Noco washout (h)

Fig. S3

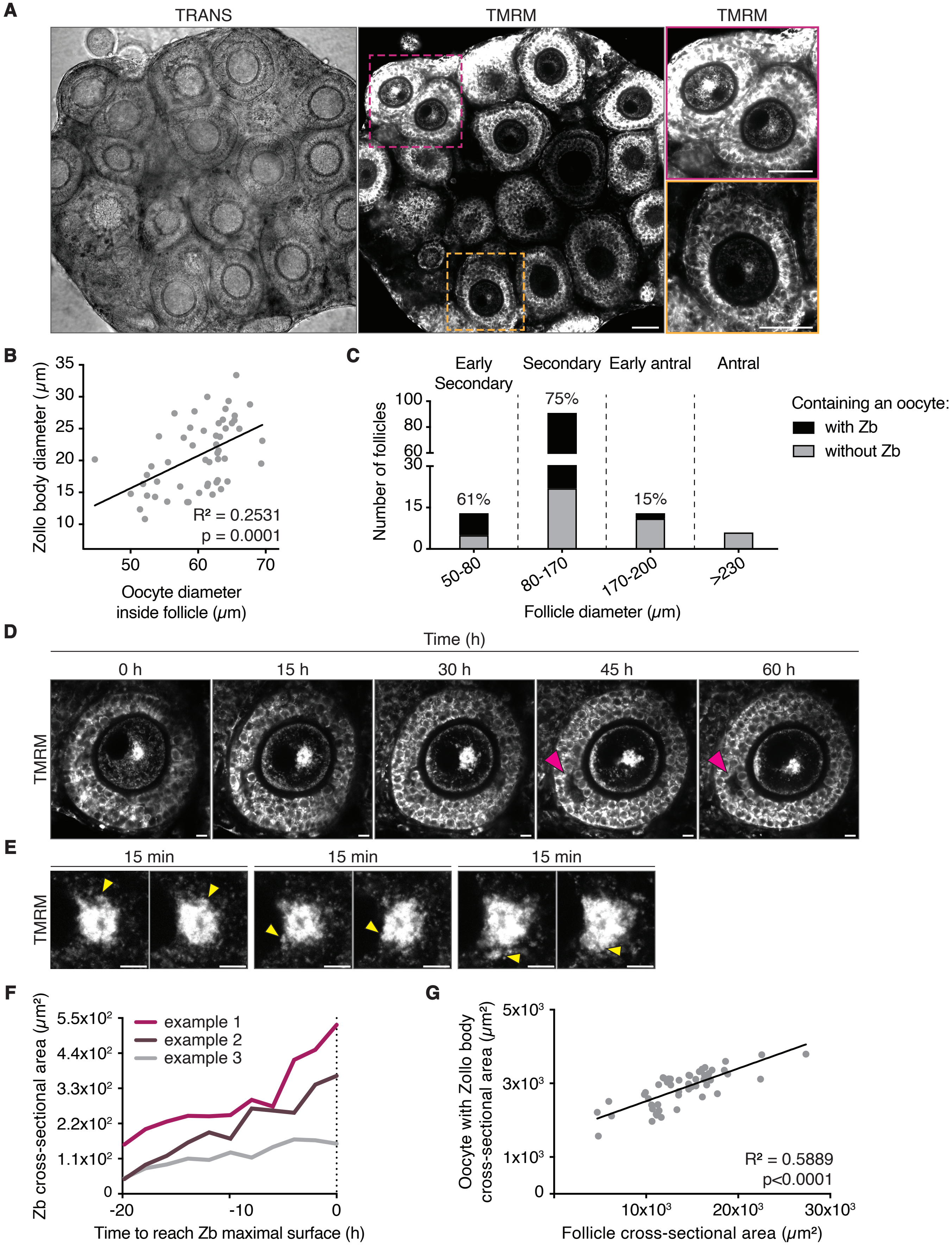
A
TRANS
TMRM
TMRM
B
Zollo body diameter (μm)
35
30
25
20
15
10
R² = 0.2531
p = 0.0001
50
60
70
Oocyte diameter inside follicle (μm)
C
Early Secondary
Secondary
Early antral
Antral
Number of follicles
61%
75%
15%
Containing an oocyte:
with Zb
without Zb
50-80
80-170
170-200
>230
Follicle diameter (μm)
D
Time (h)
0 h
15 h
30 h
45 h
60 h
TMRM
E
15 min
15 min
15 min
TMRM
F
Zb cross-sectional area (μm²)
5.5x10²
4.4x10²
3.3x10²
2.2x10²
1.1x10²
example 1
example 2
example 3
-20
-10
0
Time to reach Zb maximal surface (h)
G
Oocyte with Zollo body cross-sectional area (μm²)
5x10³
3x10³
1x10³
R² = 0.5889
p<0.0001
10x10³
20x10³
30x10³
Follicle cross-sectional area (μm²)

Fig. S4

A

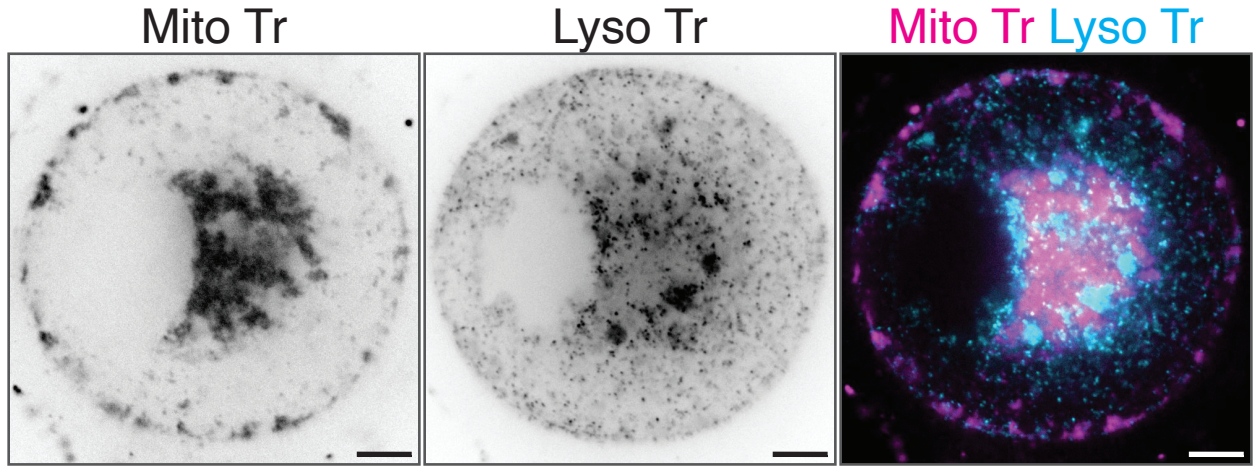
Mito Tr
Lyso Tr
Mito Tr Lyso Tr

B

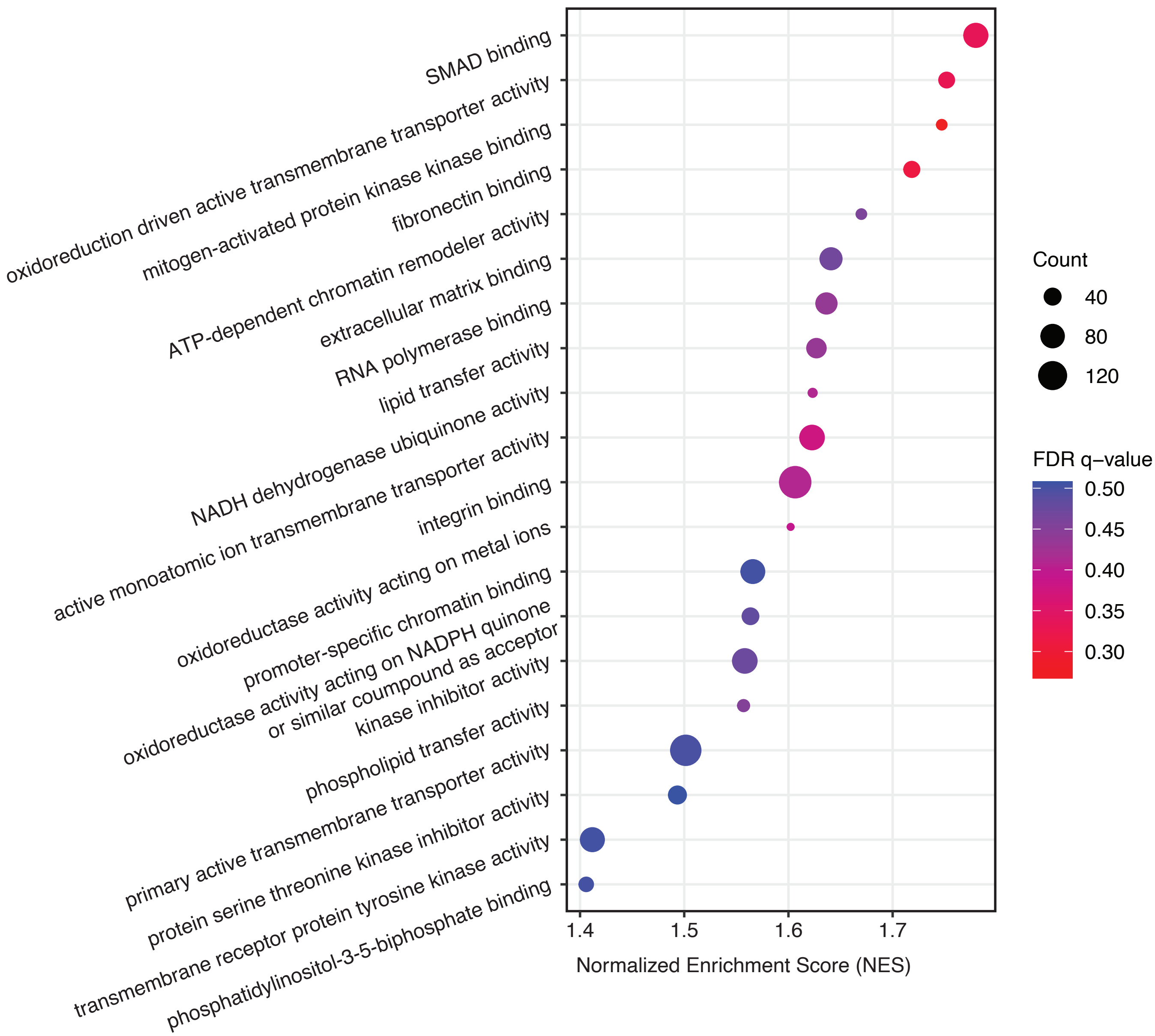
GSEA – Molecular Function
SMAD binding
oxidoreduction driven active transmembrane transporter activity
mitogen-activated protein kinase kinase binding
fibronectin binding
ATP-dependent chromatin remodeler activity
extracellular matrix binding
RNA polymerase binding
lipid transfer activity
NADH dehydrogenase ubiquinone activity
active monoatomic ion transmembrane transporter activity
integrin binding
oxidoreductase activity acting on metal ions
promoter-specific chromatin binding
oxidoreductase activity acting on NADPH quinone or similar coumpound as acceptor
kinase inhibitor activity
phospholipid transfer activity
primary active transmembrane transporter activity
protein serine threonine kinase inhibitor activity
transmembrane receptor protein tyrosine kinase activity
phosphatidylinositol-3-5-biphosphate binding
1.4
1.5
1.6
1.7
Normalized Enrichment Score (NES)
Count
40
80
120
FDR q–value
0.50
0.45
0.40
0.35
0.30

Fig. S5

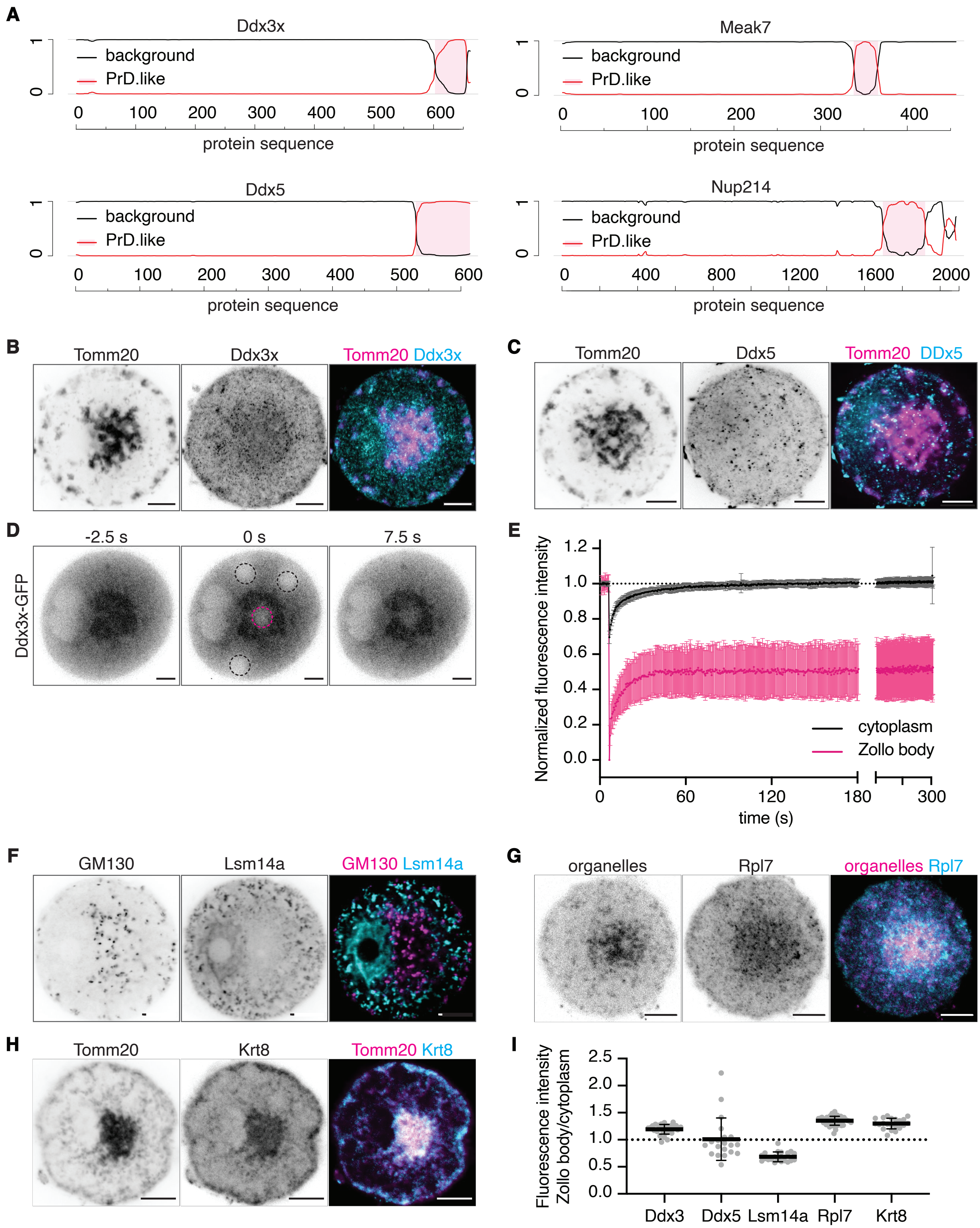
A
Ddx3x
background
PrD.like
protein sequence
Meak7
Nup214
Ddx5
B
Tomm20
Ddx3x
Tomm20 Ddx3x
C
Ddx5
Tomm20 DDx5
D
-2.5 s
0 s
7.5 s
Ddx3x-GFP
E
Normalized fluorescence intensity
cytoplasm
Zollo body
time (s)
F
GM130
Lsm14a
GM130 Lsm14a
G
organelles
Rpl7
organelles Rpl7
H
Krt8
Tomm20 Krt8
I
Fluorescence intensity
Zollo body/cytoplasm
Ddx3
Ddx5
Lsm14a
Rpl7
Krt8

Fig. S6

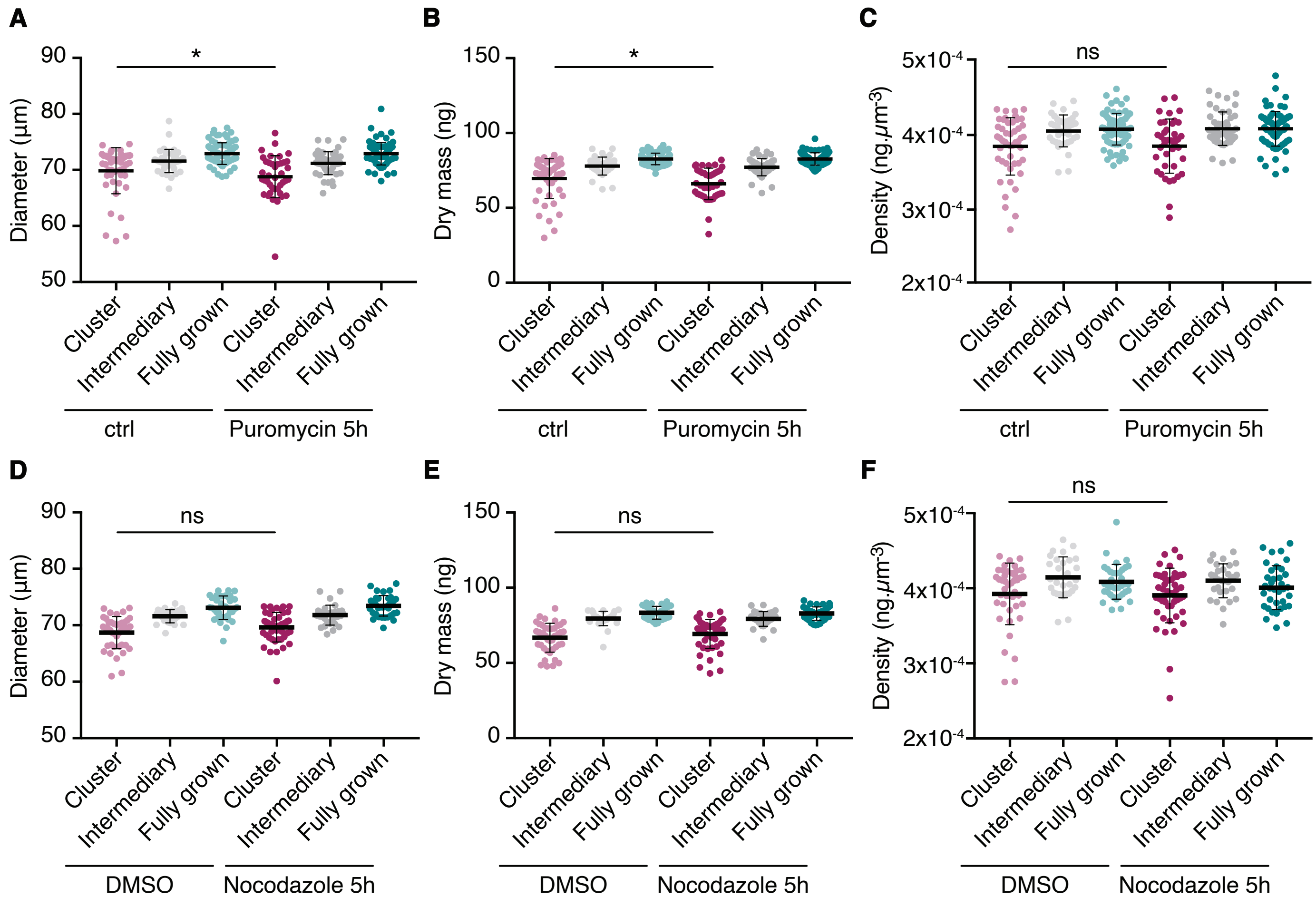
A
B
C
D
E
F
Diameter (µm)
Dry mass (ng)
Density (ng.µm-3)
*
ns
Cluster
Intermediary
Fully grown
ctrl
Puromycin 5h
DMSO
Nocodazole 5h
